# Suicidal acts and thoughts among persons with psychotic disorders in the Finnish SUPER study

**DOI:** 10.1192/j.eurpsy.2025.10066

**Published:** 2025-07-17

**Authors:** Johan Ahti, Willehard Haaki, Tuula Kieseppä, Jaana Suvisaari, Solja Niemelä, Kimmo Suokas, Minna Torniainen-Holm, Asko Wegelius, Olli Kampman, Markku Lähteenvuo, Tiina Paunio, Jari Tiihonen, Jarmo Hietala, Erkki T. Isometsä

**Affiliations:** 1 Department of Psychiatry, University of Helsinki and Helsinki University Hospital, Helsinki, Finland; 2 Department of Psychiatry, University of Turku, Turku, Finland; 3 Department of Psychiatry, Turku University Hospital, Turku, Finland; 4 Hospital District of Helsinki and Uusimaa, Helsinki, Finland; 5 Mental Health Team, Finnish Institute for Health and Welfare, Helsinki, Finland; 6 Department of Psychology, Faculty of Medicine, University of Helsinki, Helsinki, Finland; 7 Mental Health Unit, Finnish Institute for Health and Welfare, Helsinki, Finland; 8 Department of Clinical Sciences, Psychiatry, Umeå University, Umeå, Sweden; 9 Department of Clinical Medicine (Psychiatry), Faculty of Medicine, University of Turku, Turku, Finland; 10 Department of Psychiatry, The Wellbeing Services County of Ostrobothnia, Vaasa, Finland; 11 Department of Psychiatry, The Pirkanmaa Wellbeing Services County, Tampere, Finland; 12 Department of Forensic Psychiatry, Niuvanniemi Hospital, University of Eastern Finland, Kuopio, Finland; 13 SleepWell Research Program and Department of Psychiatry, Faculty of Medicine, University of Helsinki and Helsinki University Hospital; Mental Health Unit, Finnish Institute for Health and Welfare, Helsinki, Finland; 14 Department of Clinical Neuroscience, Karolinska Institutet and Center for Psychiatry Research, Stockholm City Council, Stockholm, Sweden

**Keywords:** bipolar disorder, psychotic depression, schizoaffective disorder, schizophrenia, self-harm, suicide, suicide attempt

## Abstract

**Background:**

Psychotic disorders, including schizophrenia (SZ), schizoaffective disorder (SZA), bipolar disorder (BD), psychotic depression (PD), and other nonaffective psychoses (ONAP), are associated with increased risk of suicidal acts. Few studies have compared suicidal act prevalence across psychotic disorders using both self-report and register data. The impact of hospitalization duration on subsequent suicidal acts is unclear.

**Methods:**

We used data from the SUPER-Finland study, involving 7067 participants with register-based ICD-10 diagnoses of psychotic disorders (SZ, SZA, BD, PD, ONAP). Lifetime suicidal acts were identified through self-report and register-based records of intentional self-harm events requiring medical treatment. Associations between diagnostic categories and suicidal acts were assessed using logistic regression, adjusted for sex, duration of illness, socioeconomic status, childhood abuse, and substance use. Survival analysis was used to examine the impact of hospital stay length on postdischarge self-harm.

**Results:**

Lifetime suicide attempts (39.1%) and register self-harm (19.3%) were prevalent. of those with self-reported suicide attempts, 40.5% also had register-based self-harm. Self-harm and suicide attempts were significantly more prevalent in SZA, BD, and PD compared to schizophrenia, with large differences between groups (24.1–46.4% for suicide attempts, 11.1–23.9% for self-harm). Adjusted odds of self-harm were higher for disorders with a mood component. Shorter hospitalizations were associated with an elevated hazard ratio for subsequent self-harm.

**Conclusions:**

Prevalence of register-based self-harm and self-reported suicide attempts differ markedly. Suicidal acts are common in psychotic disorders, particularly in those with a mood component. Very short inpatient stays may not be adequate in these disorders.

## Introduction

Suicidal behavior remains a major concern in psychotic disorders. Among individuals with schizophrenia (SZ), approximately 5% die by suicide, while 25–50% have at least one suicide attempt [1]. Among those with bipolar disorder (BD) or depressive disorders, 2–8% of psychiatric inpatients die by suicide in the long term [2]. A further 30–40% in major depressive disorder (MDD), and 50% in BD, attempt suicide [3]. Schizoaffective disorder (SZA), psychotic depression (PD), and other nonaffective psychoses (ONAP) are also associated with suicide risk, but are less studied [4,5]. SZA may be associated with greater risk of suicide, due to the overlap of mood and psychotic symptoms [4].

There is no universal nomenclature for suicidal behavior currently [6]. There are two primary methods of obtaining suicide attempt rates: self-report and medical records of self-harm-associated events [7]. The agreement between these methods among patients with psychotic disorders is unknown. Not all self-harm behavior is suicidal [8], and more than half of those who commit suicide die on their first attempt [3]. Nonetheless, survivors of suicide attempts provide important insights, and remain at elevated risk of suicide over time [2]. In this article, we refer to self-reported suicide attempts and register-based self-harm episodes, and use the term “suicidal acts” to encompass both suicide attempts and self-harm.

Important risk factors for suicide across diagnostic categories include adverse life events, substance use, comorbid depression, and deliberate self-harm [1, 9, 10]. While women may be more likely to attempt suicide, men are more likely to die by suicide, a pattern that holds true even in psychotic disorders [1, 2, 10–12]. Case fatality ratios differ greatly by method used [13]. Among patients admitted to psychiatric care, risk factors for suicide may differ from the general population [14]. The period immediately after discharge from inpatient care in particular increases risk for suicidal behavior [15–17]. Shorter hospital stays have been linked with greater risk of postdischarge suicide or self-harm in several studies [18–21], but other recent studies have not supported the association with suicide [17, 21–23].

This study seeks to: (1) clarify the risk of suicidal acts associated with different psychotic disorders, including schizophrenia, SZA, BD, PD, and ONAP, by assessing both register-based self-harm and self-reported suicide attempts; (2) compare these two methods of identifying suicidal acts; and (3) examine the relationship between hospitalization duration and subsequent suicidal acts in the different disorders a large clinical population. Additionally, the timing of self-harm, prevalence of violent and repeating self-harm, as well as suicidal ideation, both lifetime and in past 12 months, are reported. To clarify the association between diagnostic category and suicidal acts, demographic variables as well as questionnaire data on childhood adversity, socioeconomic status, and substance use were adjusted for.

## Materials and methods

### Study population and design

The SUPER-Finland study group recruited 10,474 people with a diagnosis of psychotic illness in Finland from primary and specialized psychiatric care, supported housing units, and the general population via newspaper advertising [24]. Participants over 18 years old and capable of informed consent gave their written informed consent.

Participants completed a questionnaire form and were interviewed in person by research nurses using a preset interview form. These data were associated with register records.

All diagnosis data used in this study were based on the Finnish Care Register for Health Care. The register includes the start and end dates of each treatment episode, ICD-10 diagnosis for each episode, medical specialty of the service provider, and ICD-10 external cause of accident where appropriate.

The Ethics Committee of the Hospital District of Helsinki and Uusimaa approved the study (reference number 202/13/03/00/15).

#### Psychotic disorders

The psychiatric diagnostic category was based on ICD-10. Participants were classified according to the most severe lifetime psychiatric diagnosis received (“major diagnosis”), in the following hierarchical order of severity: (1) SZ (F20), (2) SZA (F25), (3) BD (F30, F31), (4) PD (F32.3, F33.3), (5) ONAP (F22, F23, F24, F28, F29, F10.5, F10.75, F11.5, F12.5, F13.5, F14.5, F15.5, F16.5, F18.5, F19.5, F00.11, F00.21, F00.12, F00.22, F01.11, F01.12, F01.81, F01.82,F02.11, F02.02, F02.11, F02.12, F02.31, F02.32, F02.41, F02.42, F02.81, F02.82, F06.0, F06.2), per the SUPER study protocol [24]. For the dates of onset as defined next, first psychosis refers to the first psychiatric episode with a diagnosis of any of the previous categories, and first psychiatric diagnosis refers to any ICD-10 psychiatric diagnosis found in the records. The WHO ICD-10 Classification of Mental and Behavioral Disorders Diagnostic Criteria for Research are used for classification of psychiatric disorders in Finland [25]. The Care Register for Health Care has used ICD-10 codes since 1996 [26].

To ensure homogeneity of diagnostic criteria, the participants in this sub-study had a register-based ICD-10 diagnosis of SZ, SZA, BD, PD, or ONAP diagnosed for the first time in 1996 or later.

##### Case selection, missing values, and outlier exclusion

Of the original SUPER study population, valid consent information was available for 10,409 separate participants. Valid register-based major diagnoses set after 1996 were identified for 7460 participants which were the population for this substudy. Missing age and education data could be inferred from register data or year of birth. Any interview or questionnaire data were missing for 5.2% (*n* = 393) of these participants, with the highest number of missing responses in the drug abuse question at 3.3% (*n* = 243). Complete case (*n* = 7067) analysis was pursued. To study the effect of the date cutoff for the start of ICD-10 records, a subset of participants born after 1990, that is, aged 6 years or less at the start of record were examined separately.

### Suicidal acts

#### Self-reported suicide attempts and suicidal ideation

Participants were asked “Have you ever had suicidal thoughts?,” with the response options: never, yes during the past 12 months, and yes more than 12 months ago. They were also asked “Have you ever attempted suicide?,” with the response options: never, once, more than once, and a field for writing the number of times.

#### Register-based information on self-harm episodes

Self-harm events requiring medical attention were identified in the register data. To avoid counting repeat diagnoses of one incident (e.g., consultations from different specialties or hospital transfers) as separate incidents, self-harm diagnoses were studied by episode. An episode was defined as the period between the first date of arrival at a healthcare provider and the last date of discharge among those episodes with a date of arrival during an episode.

For all participants with a valid major diagnosis, separate episodes ending after the year 1996 and containing a ICD-10 external cause diagnosis of intentional self-harm (X60–X84) were identified (Supplementary Table S1). Intentional self-poisoning (X60–X69) was analyzed collectively regardless of the poison used and termed nonviolent self-harm, while the other diagnostic categories of self-harm were analyzed separately and collectively termed violent self-harm, in accordance with previous research [27]. Multiple self-harm refers to a participant having more than one separate self-harm episode.

#### Time intervals and psychiatric hospitalization

Participant age at the following events was calculated: first psychiatric diagnosis, first psychosis, major psychotic disorder diagnosis, and start of self-harm episode. The following time intervals were calculated: first psychiatric diagnosis to self-harm, first psychosis to self-harm, and major diagnosis to self-harm.

For each self-harm episode, the records were scanned for the previous and following psychiatric hospitalization episode, defined as a separate episode which contained at least an overnight stay at a unit specialized in psychiatry. Time from the previous hospitalization to self-harm episode was calculated. The duration of this hospitalization was calculated as defined earlier for episodes. Additionally, the previous healthcare record for an episode of any specialty was identified and time from this to the self-harm episode was calculated. The number of separate psychiatric hospitalizations had been calculated for a previous study for this sample [28] using a similar definition of separate episode.

### Background variables

#### Sociodemographic variables

The participants were interviewed using questions derived from the Finnish Health 2000 and 2011 general population surveys about their marital status and whether they have children, where a response of married, civil union, registered partnership, or common-law marriage was collectively termed “currently married or cohabiting” (short: “married”) [24]. Education level was assessed in the interview and classified according to the Health 2000 study [29]. Low level of education refers to no secondary degree after compulsory education. Sex corresponds to sex recorded the Finnish Population Information System, which is binary, and corresponds to sex assigned at birth or confirmed gender.

Participants also provided information on their employment during the last year, where working, working part-time, or studying were considered “working or studying” and on their housing status, which was classified as independent living or not living independently (e.g. supported housing) as detailed in a previous article [28].

#### Adverse childhood experiences

The participant was asked about adverse childhood experiences, with response options of yes, no, or I cannot say, including bullying at school, physical assault, and sexual abuse. Responses of yes were used in the analysis.

#### Self-reported lifetime drug use

The participant was asked about lifetime abuse of cannabis, ecstasy, amphetamine, cocaine, heroin, buprenorphine, benzodiazepines, central nervous system affecting medications, LSD, inhalants, psilocybin, and any other drugs, with the response options of: (1) not having used; (2) having used occasionally; or (3) having used more than 50 times. For this study, drug use was defined as self-reported abuse of at least one of these drugs at least occasionally. Lifetime alcohol use information was not available. Use of any of these drugs was used as a covariate in the logistic regression models. For the pairwise associations, cannabis was studied separately as the most prevalent drug of abuse.

### Statistical analysis

Prevalences were calculated for reported suicide attempts and register self-harm. Pairwise associations were also studied for presence of multiple self-reported suicide attempts, multiple register-based self-harm episodes, and violent register-based self-harm. In the survival analysis, time to event was the outcome of interest. Chi-square tests for association were performed for the association between suicidal acts, violent self-harm, multiple self-harm, suicidal thoughts in the past 12 months and the following predictors: female sex, working or studying, currently married or cohabiting, having children, low education level, living independently, reported childhood bullying, physical abuse, sexual abuse, use of cannabis, use of other drugs, and hospitalizations over median. This exploratory analysis was repeated within diagnostic groups.

Logistic regression analysis was performed for association between diagnostic category and suicidal acts, adjusting for background variables linked to suicidal acts that differed between diagnostic categories. The variables of sex, age, childhood sexual abuse, and substance use were included due to being associated with suicidal acts and their different distributions across diagnoses. Bullying and physical abuse were distributed similarly across the diagnostic categories and thus not included in the regression model. Employment status, low education level, and independent living were also included given their relevance to suicidal acts and different distribution across diagnostic groups. Having children was not found to have a pairwise association and did not improve model fit or associate with suicidal acts in preliminary analyses and was not included. Duration of illness (from first psychiatric diagnosis of any kind to start of the self-harm episode) and sex were included as covariates in model A. Additionally, independent living, low education level, employment status, sexual abuse, and drug use were included as covariates in model B. Model fit, linearity, and the inclusion of variables were assessed using Hosmer and Lemeshow’s test, generalized additive models, and Nagelkerke’s pseudo-*R* ^2^. A logarithm transformation was applied to duration of illness to improve calibration, as the relationship between duration of illness and register self-harm showed nonlinearity after 30 years.

Cox regression (proportional hazards model) was performed and Kaplan–Meier plots produced to assess survival without self-harm after hospitalization, where the population at risk consisted of all instances of discharge (*n* = 51,127) from overnight psychiatric hospitalization starting from 1996 in all study participants, with diagnostic category and hospitalization duration as predictor variables. The final model was adjusted for age at the start of follow-up, sex, education level, and childhood sexual abuse. Employment status, mode of habitation, and lifetime substance use were collected at point of interview only and could not reliably be related to the follow-up periods. A frailty model was used to adjust for repeat events [30]. The proportional hazards assumption was assessed using Schoenfeld residuals. Sensitivity analyses included testing the association between hospitalization duration and hazard within each diagnostic group separately. Follow-up started from the discharge date, and survival time was the follow-up time to a self-harm episode (event) or next psychiatric overnight hospitalization episode (if no event occurred before rehospitalization; no event) or the date of the end of the electronic record available (no event), if no rehospitalization or event occurred. Events during hospitalization or without previous hospitalization were excluded. Administrative censoring was performed at 720 days. In total, the Cox regression models included 37,074 person-years.

## Results

### Prevalence of suicidal acts and thoughts across psychotic disorders

Lifetime self-reported suicide attempts (39.1%) and self-harm events requiring medical attention (19.3%) were common among persons with psychotic disorders. However, significant variation was observed between diagnostic groups. The prevalence of suicide attempts ranged from 24.1% (ONAP) to 46.4% (SZA). Corresponding prevalence for register-based information on self-harm events ranged from 11.1% (ONAP) to 23.9% (PD). Having any of self-reported suicide attempts, suicidal ideation or register-based information on a self-harm event, was highly prevalent in all diagnostic groups ranging from 61.4% (ONAP) to 81.5% (PD) (Table 1).

**Table 1.** Suicidal acts and demographic and clinical variables in the study population (n = 7067)

Variable (*n*, % or median, IQR)	Schizophrenia (*n* = 3441)	SZA (*n* = 824)	BD (*n* = 1439)	PD (*n* = 464)	ONAP (*N* = 899)	p*
Reported suicide attempt	1291 (37.5%)	382 (46.4%)	665 (46.2%)	208 (44.8%)	217 (24.1%)	<0.0001
*…also has register SH*	487 (37.7%)	165 (43.2%)	295 (44.4%)	102 (49%)	71 (32.7%)	0.00030
Register self-harm	622 (18.1%)	189 (22.9%)	342 (23.8%)	111 (23.9%)	100 (11.1%)	<0.0001
*…also reports SA*	487 (78.3%)	165 (87.3%)	295 (86.3%)	102 (91.9%)	71 (71%)	<0.0001
Suicidal act**	1426 (41.4%)	406 (49.3%)	712 (49.5%)	217 (46.8%)	246 (27.4%)	<0.0001
Reported multiple SA	639 (18.6%)	200 (24.3%)	392 (27.2%)	118 (25.4%)	93 (10.3%)	<0.0001
*…also multiple SH ep.*	145 (22.7%)	57 (28.5%)	121 (30.9%)	35 (29.7%)	20 (21.5%)	0.029
*no. reported SA*	3 (3)	3 (3)	3 (3)	3 (3)	3 (2)	–
*no. SH episodes*	1 (1)	1 (2)	2 (2)	1 (2)	1.5 (2)	–
Multiple SH episodes	205 (6%)	71 (8.6%)	152 (10.6%)	48 (10.3%)	36 (4%)	<0.0001
*…also reports multiple SA*	145 (70.7%)	57 (80.3%)	121 (79.6%)	35 (72.9%)	20 (55.6%)	0.022
Violent SH episodes	135 (3.9%)	39 (4.7%)	54 (3.8%)	33 (7.1%)	21 (2.3%)	0.00058
*…also reports SA*	102 (75.6%)	34 (87.2%)	47 (87%)	32 (97%)	16 (76.2%)	0.028
Suicidal thoughts past 12 months	919 (26.7%)	309 (37.5%)	597 (41.5%)	234 (50.4%)	278 (30.9%)	<0.0001
Suicidal thoughts lifetime	2094 (60.9%)	611 (74.2%)	1125 (78.2%)	366 (78.9%)	523 (58.2%)	<0.0001
*…also has suicidal acts*	1239 (59.2%)	374 (61.2%)	667 (59.3%)	205 (56%)	217 (41.5%)	<0.0001
Never suicidal acts or thoughts	1160 (33.7%)	181 (22%)	269 (18.7%)	86 (18.5%)	347 (38.6%)	<0.0001
Age	41 (18)	41 (21)	44 (22)	45 (30)	36 (24.5)	–
No. hospitalizations	5 (9)	5 (7)	3 (6)	3 (5)	2 (2)	–
Female	1429 (41.5%)	517 (62.7%)	906 (63%)	288 (62.1%)	438 (48.7%)	<0.0001
Working or studying	360 (10.5%)	124 (15%)	311 (21.6%)	77 (16.6%)	224 (24.9%)	<0.0001
Low education level	1223 (35.5%)	190 (23.1%)	282 (19.6%)	130 (28%)	283 (31.5%)	<0.0001
Living independently	2281 (66.3%)	682 (82.8%)	1309 (91%)	383 (82.5%)	734 (81.6%)	<0.0001
Has children	847 (24.6%)	351 (42.6%)	845 (58.7%)	210 (45.3%)	278 (30.9%)	<0.0001
Reports bullying	1917 (55.7%)	478 (58%)	805 (55.9%)	251 (54.1%)	501 (55.7%)	0.71
Reports physical abuse	863 (25.1%)	204 (24.8%)	398 (27.7%)	125 (26.9%)	205 (22.8%)	0.091
Reports sexual abuse	439 (12.8%)	153 (18.6%)	252 (17.5%)	86 (18.5%)	111 (12.3%)	<0.0001
Used cannabis	1252 (36.4%)	303 (36.8%)	508 (35.3%)	102 (22%)	349 (38.8%)	<0.0001
Used other drugs	1160 (33.7%)	266 (32.3%)	441 (30.6%)	95 (20.5%)	280 (31.1%)	<0.0001

Abbreviations: SA, suicide attempt; SH, self-harm.

*Note:* *chi-square test of independence, **either reports suicide attempt or has register self-harm.

Overall, 49 (0.69%) individuals were identified as extreme outliers among individuals with multiple episodes (over nine self-harm episodes, median 16). They accounted for 1073 (31.9%) episodes in total. In this subpopulation there were significantly more self-harm episodes than reported suicide attempts, in contrast to the whole population, where a greater number of suicide attempts are reported than self-harm episodes. Where noted next, statistical tests were run with and without these outliers. Summary statistics for the outliers are reported in the Supplementary Materials.

Of those reporting suicide attempts, a minority (40.5%) had register-based self-harm episodes. Of those with register-based self-harm episode, a majority (82.1%) reported suicide attempts. The proportion of suicide attempts associated with self-harm was higher in SZA, BD, and PD than in SZ or ONAP. In the subgroup born after 1990, similarly, a minority (48.1%) of those reporting suicide attempts had register-based self-harm (reported in the Supplementary Materials).

Results for the exploratory pairwise chi-square tests of association between the background variables and suicidal acts are shown in Table 2. The tests of association within diagnostic groups as well as with suicidal thoughts, and associations with violent and multiple self-harm episodes are reported in the Supplementary Materials.

**Table 2.** Pairwise associations between background variables, suicide attempts, and self-harm

	Suicide attempts				Register self-harm			
Participants	Factor present	Factor absent	χ^2^	*p*	Factor present	Factor absent	χ^2^	*p*
All (*n* = 7067)	2763 (39.1%)	–	–	–	1364 (19.3%)	–	–	–
Female (*n* = 3578)	1551 (43.3%)	1212 (34.7%)	54.64	<0.0001	778 (21.7%)	586 (16.8%)	27.45	<0.0001
Not working or studying (*n* = 5971)	2431 (40.7%)	332 (30.3%)	41.8	<0.0001	1226 (20.5%)	138 (12.6%)	36.99	<0.0001
No children (*n* = 4536)	1729 (38.1%)	1034 (40.9%)	4.993	0.025	846 (18.7%)	518 (20.5%)	3.322	0.068
Low education level (*n* = 2108)	926 (43.9%)	1837 (37%)	29.15	<0.0001	529 (25.1%)	835 (16.8%)	64.22	<0.0001
Not living independently (*n* = 1678)	761 (45.4%)	2002 (37.1%)	35.81	<0.0001	430 (25.6%)	934 (17.3%)	55.98	<0.0001
Bullied (*n* = 3952)	1742 (44.1%)	1021 (32.8%)	92.97	<0.0001	821 (20.8%)	543 (17.4%)	12.28	<0.0001
Physical abuse (*n* = 1795)	943 (52.5%)	1820 (34.5%)	181.7	<0.0001	415 (23.1%)	949 (18%)	22.2	<0.0001
Sexual abuse (*n* = 1041)	629 (60.4%)	2134 (35.4%)	232.1	<0.0001	285 (27.4%)	1079 (17.9%)	50.52	<0.0001
Cannabis use (*n* = 2514)	1203 (47.9%)	1560 (34.3%)	125	<0.0001	670 (26.7%)	694 (15.2%)	134.6	<0.0001
Use of other drugs (*n* = 2242)	1174 (52.4%)	1589 (32.9%)	241.9	<0.0001	679 (30.3%)	685 (14.2%)	253.3	<0.0001
Hospitalizations over median (*n* = 3266)	1717 (52.6%)	1046 (27.5%)	462	<0.0001	1029 (31.5%)	335 (8.8%)	579.3	<0.0001

**Figure 1.**
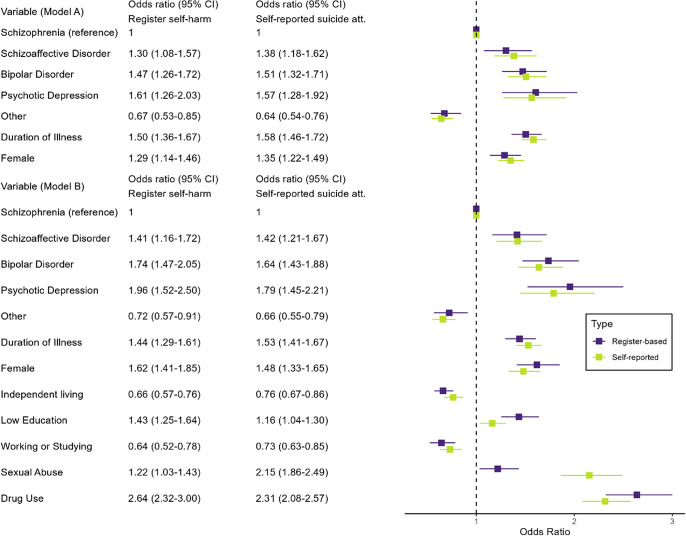
Odds ratios for the presence of at least one register-based self-harm episode and for at least *one self-reported suicide attempt by diagnostic category.* The reference category for diagnosis is schizophrenia. Model A is adjusted for duration of illness and sex, while model B is additionally adjusted for the other background factors.

Adjusting for the background factors, the associations between diagnostic category and suicide attempts were broadly similar to the associations between the diagnostic category and self-harm. Of the background variables, sexual abuse was more associated with suicide attempts than self-harm. The results of the logistic regression analyses are presented in Figure 1.

Self-harm episodes had a median between 20 and 30 years of age and left-skewed distributions, except in bipolar disorder where the distribution was wider with a median of 35.3 years of age. The distribution peak was mostly within 1 year of the first occurrence of the major diagnosis. A healthcare episode preceded 80.8% of all self-harm episodes; the healthcare episode was on median within 1 week of the self-harm episode. Summary statistics about self-harm methods and participant age at the start of the self-harm episode are shown in the Supplementary Materials.

### Length of hospitalization

**Figure 2.**
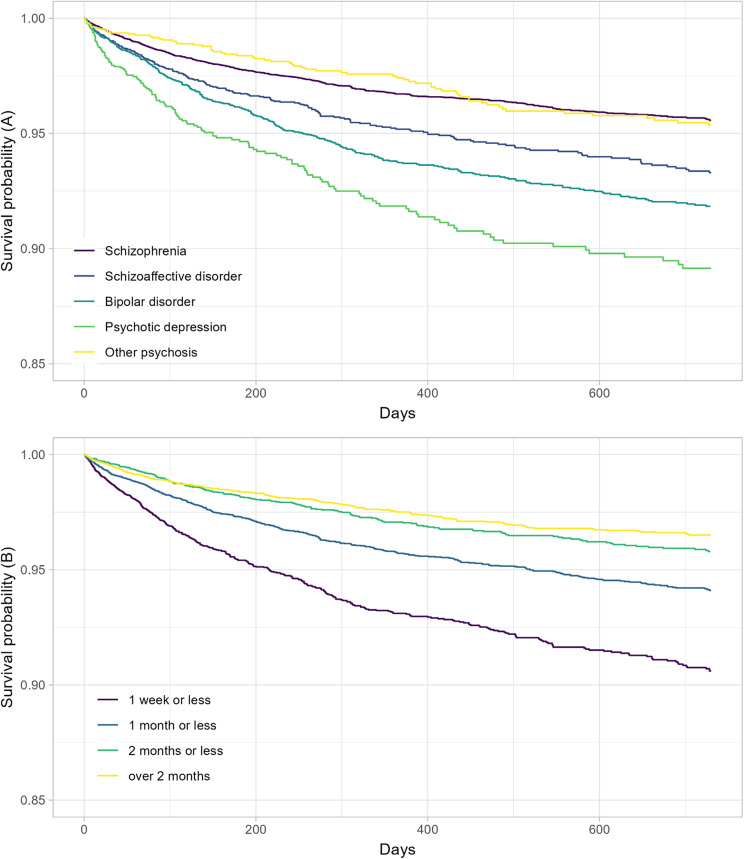
Survival without self-harm after hospitalization, by (A) hospitalization duration and (B) diagnostic category (to 2 years).

Follow-up to 2 years without a self-harm episode was significantly less likely for shorter hospitalizations. Kaplan–Meier curves without frailty from the survival analysis are presented in Figure 2. Results for the main Cox regression model are presented in Table 3. Results for the survival analysis within diagnostic categories and without the outliers are presented in the Supplementary Materials. The outliers were found to not affect the results of the Cox regression. The relationship between hospitalization duration and self-harm hazard was present in all diagnostic categories except ONAP.

**Table 3.** Cox proportional hazards model of self-harm after discharge from psychiatric hospitalization, up to 720 days of follow-up

Variable	Hazard ratio	95% CI	*p*
Diagnosis			
Schizophrenia (reference)	1		–
Schizoaffective disorder	1.43	1.15–1.76	0.0010
Bipolar disorder	1.79	1.48–2.16	<0.0001
Psychotic depression	2.83	2.16–3.70	<0.0001
Other psychosis	0.85	0.63–1.14	0.28
Hospitalization duration			
Over 2 months (reference)	1		–
2 months or less	1.18	0.96–1.45	0.13
1 month or less	1.59	1.33–1.89	<0.0001
1 week or less	2.24	1.88–2.67	<0.0001
Covariates			
Female sex	1.78	1.53–2.08	<0.0001
Low education	1.53	1.32–1.78	<0.0001
Age at discharge	0.97	0.96–0.97	<0.0001
Sexual abuse	1.19	1.00–1.42	0.052

## Discussion

### Register-based self-harm events versus self-reported suicide attempts

Most participants with suicide attempts would not have been identified by surveying register-based self-harm. Previous research in Finland has found that only approximately two thirds of those with bipolar disorder [31] and depression [32] were referred to emergency medical care; most did not communicate their attempt to healthcare personnel. In our study population, less than half (32.7–49.0%) of those who reported suicide attempts had a recorded diagnosis of intentional self-harm. This indicates that a survey of rates of suicide attempts based on register diagnoses would significantly underestimate the frequency of suicide attempts among persons with psychotic disorders. Most participants with register-based self-harm (82.1%) reported suicide attempts. The prevalence of suicidal acts is correspondingly slightly higher than self-reported suicide attempts.

Results for the logistic regression analysis were broadly similar regardless of which outcome variable was used. So, while understating the proportion with suicide attempts, register-based self-harm seemed to act as a valid proxy for the positive presence of suicide attempts. However, the associations between childhood sexual abuse, self-harm, and suicide attempts were notably different. One explanation could be differences in help seeking in the context of suicidal behavior.

### Differences between disorders

Suicidal acts and thoughts were common across the studied psychotic disorders. Those with a mood component – SZA, BD, and PD – were associated with a particularly high prevalence of self-harm and for reported suicide attempts. This association remained after adjusting for background factors. Clinical differences, in particular, depressed mood [1,3], may account for the difference between disorders. In schizophrenia, negative symptoms and poor cognitive functioning may paradoxically reduce suicidal ideation and self-harm risk due to a reduced capacity for emotional distress and ability to execute a suicide attempt [33], and lack of insight is associated with less depression [34]. Indeed, prevalence of suicidal acts were similar among those who had suicidal thoughts across schizophrenia, SZA, BD, and PD.

Schizophrenia, PD, and ONAP associated with violent self-harm, with the highest proportion of violent self-harm in PD. PD has recently been associated with violent suicide over nonpsychotic depression [5]. The hazard ratio for subsequent self-harm was higher in schizophrenia, SZA, PD, and BD after a hospitalization lasting less than 1 week. This association did not hold in ONAP. Short hospitalizations could be more appropriate in this group, which includes brief acute psychoses.

### Duration of hospitalization

We found a short hospitalization was associated with a significantly higher hazard ratio for self-harm. This agrees with a recent large cohort study, which found an association between self-harm and shorter length of stay [21]. However, several recent studies have reported no association with suicide, including a recent comprehensive cohort after hospitalization for depression in Finland [17, 21–23].

Based on the questionnaire, a significant proportion of the self-harm episodes involved repeated, predominantly nonsuicidal self-harm. Therefore, and in light of the previous literature, the findings may not translate to suicide risk.

Hospital discharge reduces supervision; shorter stays may reflect unplanned discharge or limit safety planning. While short hospitalizations are desirable for many reasons, risk of self-harm could be an adequate reason to recommend a longer period of treatment. A very short hospitalization could be insufficient for preventing self-harm in the context of acute exacerbation of psychotic disorders.

### Study strengths and weaknesses

The strengths of this study were a large population of participants with different, reliable register-based, and clearly defined diagnoses of schizophrenia, SZA, BD, PD, and ONAP, with a large number of self-harm episodes and previous hospitalizations, as well as questionnaire information not usually available in a register-based study, allowing us to compare register-based self-harm with self-reported suicide attempts and account for socioeconomic disadvantage, drug abuse, and childhood abuse. We can therefore report reliable comparisons between the disorders and adjust for potentially confounding variables.

The main weakness of this study is the sampling strategy, which is a sample of convenience mainly from healthcare settings. Therefore, our results are likely generalizable to the clinical rather than the general population. Regarding suicide prevention, suicidal acts are different from suicides, and we could not evaluate whether any given self-harm episode was a suicide attempt. To participate in the SUPER study, a participant had to survive their suicide attempt. Therefore, the results reflect history of self-harm over a long period of illness rather than risk of suicide at illness onset. Finally, the cross-sectional and register-based study design did not permit examining any given self-harm episode or contributing factors in detail, such as depressed mood or psychotic symptoms. The relationships between the predictors and response variables therefore represent associations only.

## Conclusions

Register-based self-harm was found for a minority of those with suicide attempts in this population. The disparity has implications for suicide research. The majority of those with SZ, SZA, BD, or PD had suicidal acts or thoughts, with significant between-groups differences. Psychotic disorders with a mood component, including schizoaffective disorder and psychotic depression, are associated with somewhat more suicidal acts than schizophrenia in the clinical population. Hospitalizations less than 1 week were associated with subsequent self-harm in schizophrenia, schizoaffective disorder, and psychotic mood disorders. Very short hospitalizations could be inadequate for preventing self-harm in these disorders, though adequate in brief psychotic disorders.

## Supporting information

10.1192/j.eurpsy.2025.10066.sm001 Ahti et al. supplementary material Ahti et al. supplementary material

## Data Availability

The SUPER-Finland website can be accessed for further information (https://www.superfinland.fi/english). The data from SUPER-Finland participants who gave biobank consent can be acquired from the THL Biobank when released from the original study (https://thl.fi/en/web/thl-biobank).

## Supplementary material

The supplementary material for this article can be found at http://doi.org/10.1192/j.eurpsy.2025.10066.

## References

[1] Cassidy RM, Yang F, Kapczinski F, Passos IC. Risk factors for Suicidality in patients with schizophrenia: A systematic review, meta-analysis, and meta-regression of 96 studies. Schizophr Bull. 2018;44:787–97. 10.1093/SCHBUL/SBX131. 29036388 PMC6007264

[2] Isometsä ET. Suicides in mood disorders in psychiatric settings in Nordic National Register–based studies. Front Psych. 2020;11:540285. 10.3389/FPSYT.2020.00721. PMC7390882 32848909

[3] Isometsä E. Suicidal behaviour in mood disorders-who, when, and why? Can J Psychiatr. 2014;59:120–30. 10.1177/070674371405900303. PMC4079239 24881160

[4] Álvarez A, Guàrdia A, González-Rodríguez A, Betriu M, Palao D, Monreal JA, et al. A systematic review and meta-analysis of suicidality in psychotic disorders: Stratified analyses by psychotic subtypes, clinical setting and geographical region. Neurosci Biobehav Rev. 2022;143:104964. 10.1016/J.NEUBIOREV.2022.104964. 36403792

[5] Paljärvi T, Tiihonen J, Lähteenvuo M, Tanskanen A, Fazel S, Taipale H. Psychotic depression and deaths due to suicide. J Affect Disord. 2023;321:28–32. 10.1016/J.JAD.2022.10.035 36280195

[6] Silverman MM, De Leo D Why there is a need for an international nomenclature and classification system for suicide. Crisis. 2016;37:83–7. 10.1027/0227-5910/A000419. 27232426

[7] World Health Organization. Preventing suicide: A global imperative. Geneva: World Health Organization; 2014.

[8] Goodfellow B, Kõlves K, De Leo D. Contemporary classifications of suicidal Behaviors: A systematic literature review. Crisis. 2020;41:179–86. 10.1027/0227-5910/A000622. 31512927

[9] Favril L, Yu R, Uyar A, Sharpe M, Fazel S. Risk factors for suicide in adults: Systematic review and meta-analysis of psychological autopsy studies. Evid Based Ment Health. 2022;25:148–55. 10.1136/EBMENTAL-2022-300549. 36162975 PMC9685708

[10] Nordentoft M, Mortensen PB, Pedersen CB. Absolute risk of suicide after first hospital contact in mental disorder. Arch Gen Psychiatry. 2011;68:1058–64. 10.1001/ARCHGENPSYCHIATRY.2011.113. 21969462

[11] Naghavi M. Global, regional, and national burden of suicide mortality 1990 to 2016: Systematic analysis for the global burden of disease study 2016. BMJ. 2019;364:l94. 10.1136/BMJ.L94. 31339847 PMC6598639

[12] Hu FH, Jia YJ, Zhao DY, Fu XL, Zhang WQ, Tang W, et al. Gender differences in suicide among patients with bipolar disorder: A systematic review and meta-analysis. J Affect Disord. 2023;339:601–14. 10.1016/J.JAD.2023.07.060. 37467799

[13] Cai Z, Junus A, Chang Q, Yip PSF. The lethality of suicide methods: A systematic review and meta-analysis. J Affect Disord. 2022;300:121–9. 10.1016/J.JAD.2021.12.054. 34953923

[14] Large M, Sharma S, Cannon E, Ryan C, Nielssen O. Risk factors for suicide within a year of discharge from psychiatric hospital: A systematic meta-analysis. Aust N Z J Psychiatry. 2011;45:619–28. 10.3109/00048674.2011.590465. 21740345

[15] Chung DT, Ryan CJ, Hadzi-Pavlovic D, Singh SP, Stanton C, Large MM. Suicide rates after discharge from psychiatric facilities: A systematic review and meta-analysis. JAMA Psychiatry. 2017;74:694–702. 10.1001/JAMAPSYCHIATRY.2017.1044. 28564699 PMC5710249

[16] Madsen T, Erlangsen A, Hjorthøj C, Nordentoft M. High suicide rates during psychiatric inpatient stay and shortly after discharge. Acta Psychiatr Scand. 2020;142:355–65. 10.1111/ACPS.13221. 32715465

[17] Aaltonen K, Sund R, Hakulinen C, Pirkola S, Isometsä E. Variations in suicide risk and risk factors after hospitalization for depression in Finland, 1996-2017. JAMA Psychiatry. 2024;81:506–15. 10.1001/JAMAPSYCHIATRY.2023.5512. 38353967 PMC10867776

[18] Tseng MCM, Chang CH, Liao SC, Yeh YC. Length of stay in relation to the risk of inpatient and post-discharge suicides: A national health insurance claim data study. J Affect Disord. 2020;266:528–33. 10.1016/J.JAD.2020.02.014. 32056922

[19] Qin P, Nordentoft M. Suicide risk in relation to psychiatric hospitalization: Evidence based on longitudinal registers. Arch Gen Psychiatry. 2005;62:427–32. 10.1001/ARCHPSYC.62.4.427. 15809410

[20] Bickley H, Hunt IM, Windfuhr K, Shaw J, Appleby L, Kapur N. Suicide within two weeks of discharge from psychiatric inpatient care: A case-control study. Psychiatr Serv. 2013;64:653–9. 10.1176/APPI.PS.201200026. 23545716

[21] Mortier P, Conde S, Alayo I, Amigo F, Ballester L, Cirici Amell R, et al. Premature death, suicide, and nonlethal intentional self-harm after psychiatric discharge. JAMA Netw Open. 2024;7:e2417131. 10.1001/JAMANETWORKOPEN.2024.17131. 38922620 PMC11208976

[22] König D, Gleiss A, Vyssoki B, Harrer C, Trojer A, Groemer M, et al. Suicide risk after discharge from in-patient psychiatric care: A 15-year retrospective cohort study of individual patient data. J Affect Disord. 2024;354:416–23. 10.1016/J.JAD.2024.03.046. 38479514

[23] Olfson M, Wall M, Wang S, Crystal S, Liu SM, Gerhard T, et al. Short-term suicide risk after psychiatric hospital discharge. JAMA Psychiatry. 2016;73:1119. 10.1001/JAMAPSYCHIATRY.2016.2035. 27654151 PMC8259698

[24] Lähteenvuo M, Ahola-Olli A, Suokas K, Holm M, Misiewicz Z, Jukuri T, et al. Cohort profile: SUPER-Finland - the Finnish study for hereditary mechanisms of psychotic disorders. BMJ Open. 2023;13:e070710. 10.1136/bmjopen-2022-070710. PMC10106053 37045567

[25] Komulainen J, Lehtonen J, Mäkelä M. Psykiatrian luokituskäsikirja: Suomalainen tautiluokitus ICD-10:n psykiatriaan liittyvät koodit. THL; 2012.

[26] Sund R. Quality of the Finnish hospital discharge register: A systematic review. Scand J Public Health. 2012;40:505–15. 10.1177/1403494812456637. 22899561

[27] Ludwig B, Dwivedi Y. The concept of violent suicide, its underlying trait and neurobiology: A critical perspective. Eur Neuropsychopharmacol. 2017;28:243. 10.1016/J.EURONEURO.2017.12.001. 29254658 PMC5809305

[28] Ahti J, Kieseppä T, Suvisaari J, Suokas K, Holm M, Wegelius A, et al. Differences in psychosocial functioning between psychotic disorders in the Finnish SUPER study. Schizophr Res 2022;244:10–7. 10.1016/J.SCHRES.2022.04.008. 35537381

[29] Aromaa A, Koskinen S. Health and functional capacity in Finland : Baseline results of the health 2000 health examination survey. Helsinki: National Public Health Institute = Kansanterveyslaitos; KTL; 2004.

[30] Amorim LDAF, Cai J. Modelling recurrent events: A tutorial for analysis in epidemiology. Int J Epidemiol. 2015;44:324–33. 10.1093/IJE/DYU222. 25501468 PMC4339761

[31] Valtonen HM, Suominen K, Mantere O, Leppämäki S, Arvilommi P, Isometsä ET. Prospective study of risk factors for attempted suicide among patients with bipolar disorder. Bipolar Disord. 2006;8:576–85. 10.1111/J.1399-5618.2006.00341.X. 17042831

[32] Riihimäki K, Vuorilehto M, Melartin T, Haukka J, Isometsä E. Incidence and predictors of suicide attempts among primary-care patients with depressive disorders: A 5-year prospective study. Psychol Med. 2014;44:291–302. 10.1017/S0033291713000706. 23570583

[33] Grover LE, Jones R, Bass NJ, McQuillin A. The differential associations of positive and negative symptoms with suicidality. Schizophr Res. 2022;248:42–9. 10.1016/j.schres.2022.07.016. 35933743

[34] Murri MB, Amore M, Calcagno P, Respino M, Marozzi V, Masotti M, et al. The “insight paradox” in schizophrenia: Magnitude, moderators and mediators of the association between insight and depression. Schizophr Bull. 2016;42:1225 10.1093/SCHBUL/SBW040. 27069064 PMC4988746

