# Supplementary material for: Suicidal acts and thoughts among persons with psychotic disorders in the Finnish SUPER study
Source: Eur Psychiatry. 2025 Jul 17;68(1):e99. doi: 10.1192/j.eurpsy.2025.10066 (PMC12344466; doi:10.1192/j.eurpsy.2025.10066)
Supplement: Ahti et al. supplementary material [file S0924933825100667sup001.docx]

Supplementary data

*Supplementary figure 1. Temporal relationship between events of interest and registry-based self-harm episodes. Left: without outlier exclusion, right: with outlier exclusion*

*
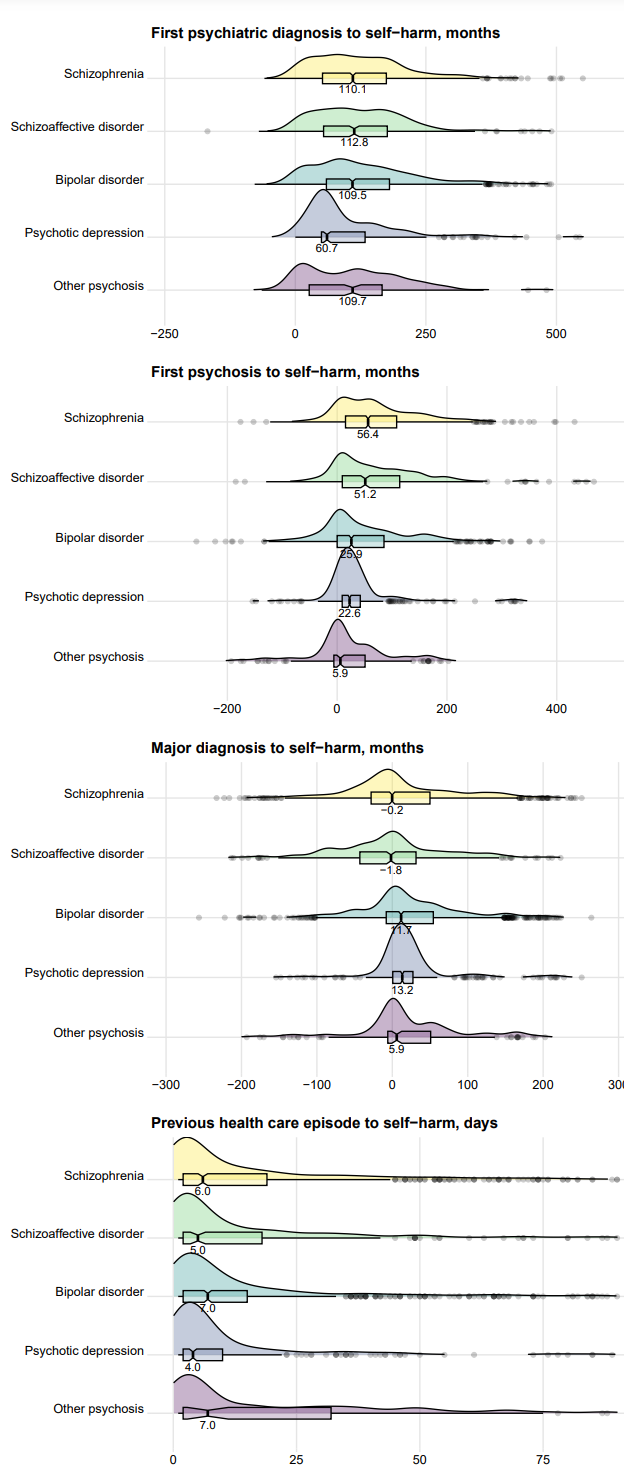
* *
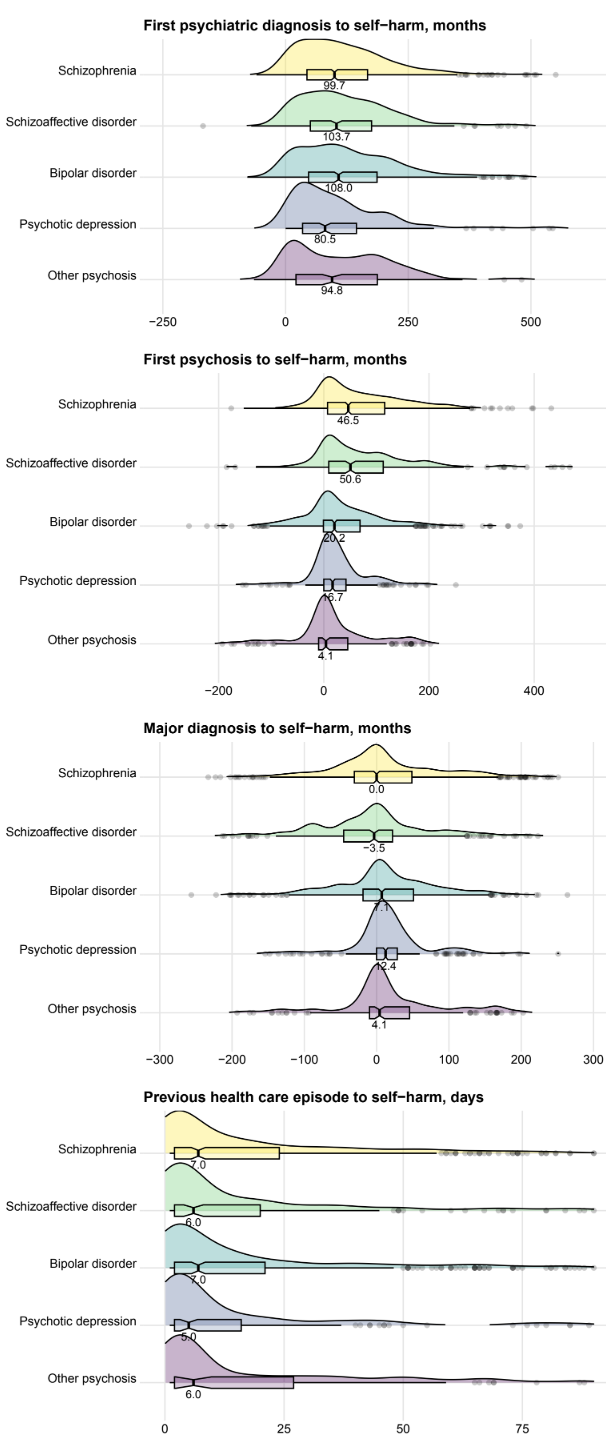
Note: a previous health care episode was not identified for 645 out of 3365 episodes (19.2 %).*

Supplementary figure 2. Survival by diagnostic category.


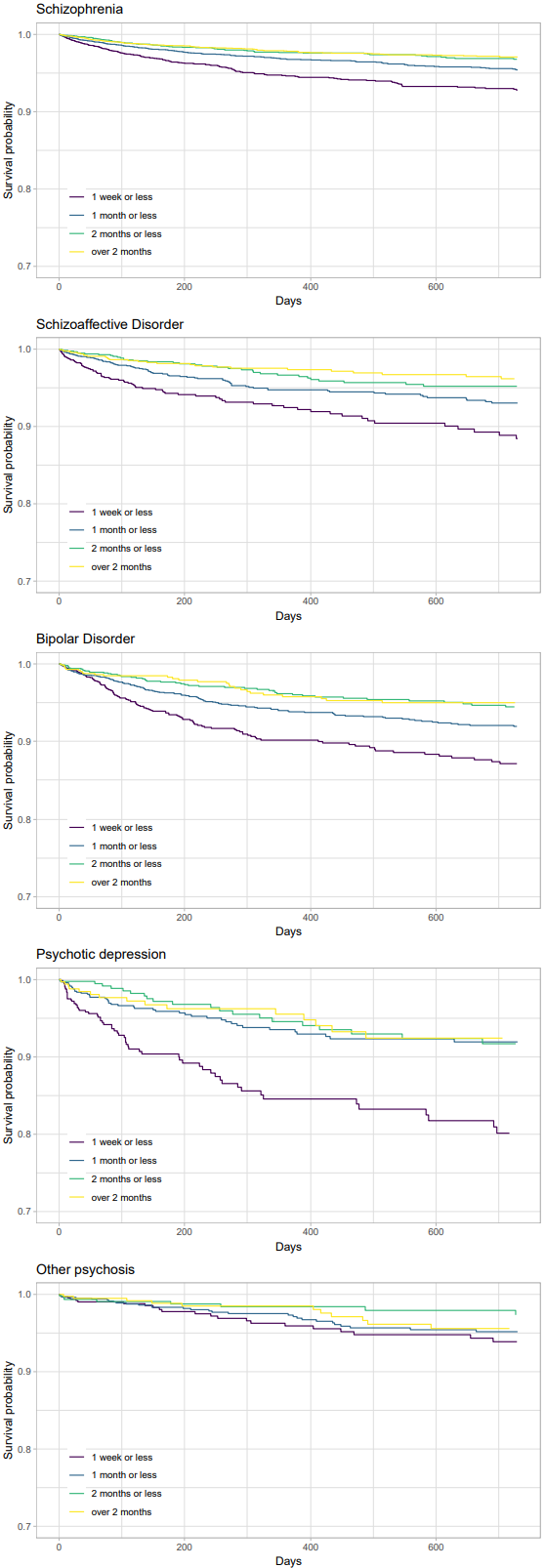


Supplementary figure 3.  *Distribution of self-harm episodes by age (top: without outlier exclusion, bottom: with outlier exclusion).*


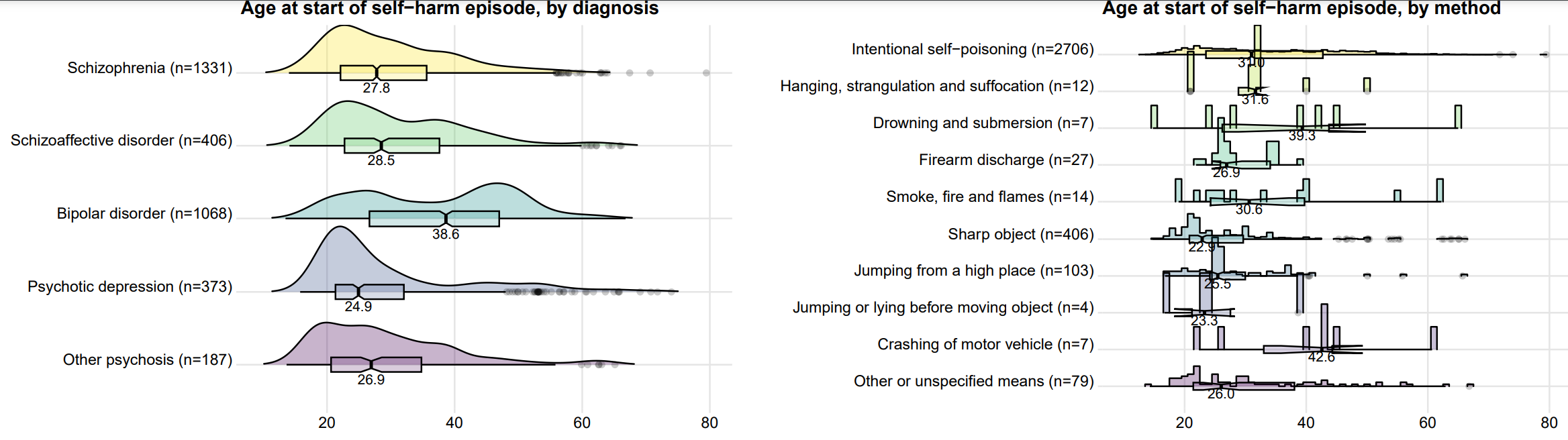


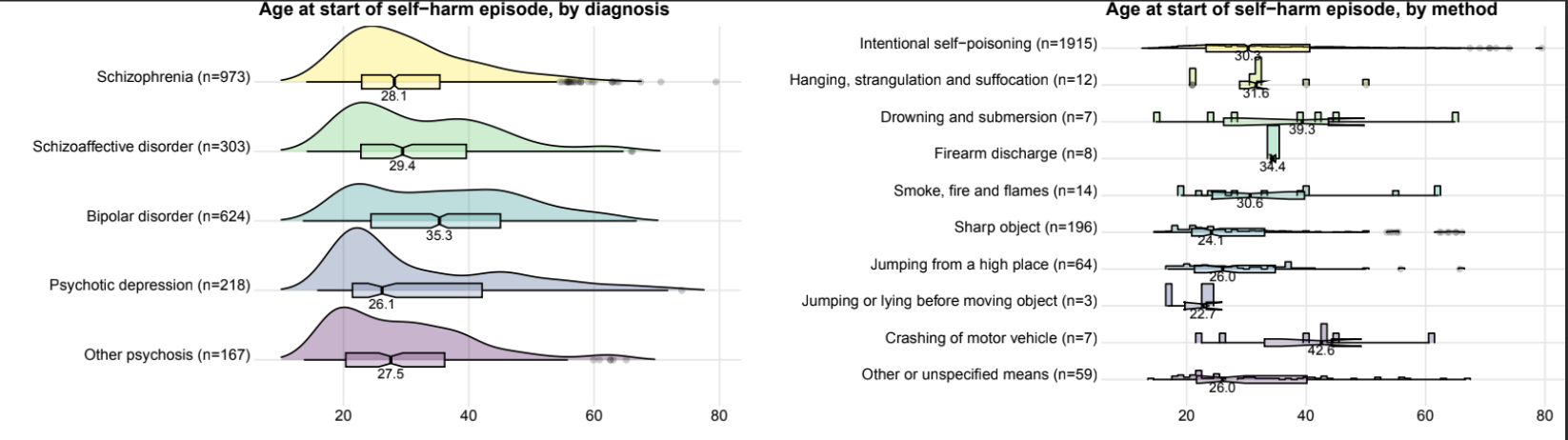


| Supplementary table 1. ICD-10 codes, definitions [CITE ICD-10] and classification | | | |
| --- | --- | --- | --- |
| Code | Definition (ICD-10) | Classification | Collectively |
| X60 | Intentional self-poisoning by and exposure to nonopioid analgesics, antipyretics and antirheumatics | Intentional self-poisoning | Non-violent self-harm |
| X61 | Intentional self-poisoning by and exposure to nonopioid analgesics, antipyretics and antirheumatics |  |  |
| X62 | Intentional self-poisoning by and exposure to narcotics and psychodysleptics [hallucinogens], not elsewhere classified |  |  |
| X63 | Intentional self-poisoning by and exposure to other drugs acting on the autonomic nervous system |  |  |
| X64 | Intentional self-poisoning by and exposure to other and unspecified drugs, medicaments and biological substances |  |  |
| X65 | Intentional self-poisoning by and exposure to alcohol |  |  |
| X66 | Intentional self-poisoning by and exposure to organic solvents and halogenated hydrocarbons and their vapours |  |  |
| X67 | Intentional self-poisoning by and exposure to carbon monoxide and other gases and vapours |  |  |
| X68 | Intentional self-poisoning by and exposure to pesticides |  |  |
| X69 | Intentional self-poisoning by and exposure to other and unspecified chemicals and noxious substances |  |  |
| X70 | Intentional self-harm by hanging, strangulation and suffocation | Considered separately by diagnosis | Violent self-harm |
| X71 | Intentional self-harm by drowning and submersion |  |  |
| X72 | Intentional self-harm by handgun discharge |  |  |
| X73 | Intentional self-harm by rifle, shotgun and larger firearm discharge |  |  |
| X74 | Intentional self-harm by other and unspecified firearm discharge |  |  |
| X75 | Intentional self-harm by explosive material |  |  |
| X76 | Intentional self-harm by smoke, fire and flames |  |  |
| X77 | Intentional self-harm by steam, hot vapours and hot objects |  |  |
| X78 | Intentional self-harm by sharp object |  |  |
| X79 | Intentional self-harm by blunt object |  |  |
| X80 | Intentional self-harm by jumping from a high place |  |  |
| X81 | Intentional self-harm by jumping or lying before moving object |  |  |
| X82 | Intentional self-harm by crashing of motor vehicle |  |  |
| X83 | Intentional self-harm by other specified means | Collectively intentional self-harm by other or unspecified means |  |
| X84 | Intentional self-harm by unspecified means |  |  |

Supplementary table 2. Pairwise associations.

| **Description** | **Reports suicide attempt** | | | |
| --- | --- | --- | --- | --- |
| Participants | Factor present | Factor absent | statistic | p-value |
| All (n=7067) | 2763 (39.1 %) |  |  |  |
| Female=TRUE (n=3578) | 1551 (43.3 %) | 1212 (34.7 %) | 54.64 | 1.40E-13 |
| Working or studying=FALSE (n=5971) | 2431 (40.7 %) | 332 (30.3 %) | 41.8 | 1.00E-10 |
| Has children=FALSE (n=4536) | 1729 (38.1 %) | 1034 (40.9 %) | 4.993 | 0.025 |
| Low education level=TRUE (n=2108) | 926 (43.9 %) | 1837 (37 %) | 29.15 | 6.70E-08 |
| Living unsupervised=FALSE (n=1678) | 761 (45.4 %) | 2002 (37.1 %) | 35.81 | 2.20E-09 |
| Reports bullying=TRUE (n=3952) | 1742 (44.1 %) | 1021 (32.8 %) | 92.97 | 5.30E-22 |
| Reports physical abuse=TRUE (n=1795) | 943 (52.5 %) | 1820 (34.5 %) | 181.7 | 2.00E-41 |
| Reports sexual abuse=TRUE (n=1041) | 629 (60.4 %) | 2134 (35.4 %) | 232.1 | 2.10E-52 |
| Used cannabis=TRUE (n=2514) | 1203 (47.9 %) | 1560 (34.3 %) | 125 | 5.00E-29 |
| Used other drugs=TRUE (n=2242) | 1174 (52.4 %) | 1589 (32.9 %) | 241.9 | 1.50E-54 |
| Hospitalizations over median=TRUE (n=3266) | 1717 (52.6 %) | 1046 (27.5 %) | 462 | 1.80E-102 |
| Schizophrenia (n=3441) | 1291 (37.5 %) |  |  |  |
| Female=TRUE (n=1429) | 618 (43.2 %) | 673 (33.4 %) | 33.8 | 6.10E-09 |
| Working or studying=FALSE (n=3081) | 1181 (38.3 %) | 110 (30.6 %) | 7.986 | 0.0047 |
| Has children=FALSE (n=2594) | 955 (36.8 %) | 336 (39.7 %) | 2.098 | 0.15 |
| Low education level=TRUE (n=1223) | 521 (42.6 %) | 770 (34.7 %) | 20.57 | 5.80E-06 |
| Living unsupervised=FALSE (n=1160) | 510 (44 %) | 781 (34.2 %) | 30.62 | 3.10E-08 |
| Reports bullying=TRUE (n=1917) | 801 (41.8 %) | 490 (32.2 %) | 33.19 | 8.40E-09 |
| Reports physical abuse=TRUE (n=863) | 420 (48.7 %) | 871 (33.8 %) | 60.45 | 7.60E-15 |
| Reports sexual abuse=TRUE (n=439) | 254 (57.9 %) | 1037 (34.5 %) | 87.82 | 7.20E-21 |
| Used cannabis=TRUE (n=1252) | 556 (44.4 %) | 735 (33.6 %) | 39.4 | 3.40E-10 |
| Used other drugs=TRUE (n=1160) | 562 (48.4 %) | 729 (32 %) | 88.48 | 5.10E-21 |
| Hospitalizations over median=TRUE (n=1922) | 927 (48.2 %) | 364 (24 %) | 212.1 | 4.70E-48 |
| Schizoaffective disorder (n=824) | 382 (46.4 %) |  |  |  |
| Female=TRUE (n=517) | 242 (46.8 %) | 140 (45.6 %) | 0.06937 | 0.79 |
| Working or studying=FALSE (n=700) | 348 (49.7 %) | 34 (27.4 %) | 20.17 | 7.10E-06 |
| Has children=FALSE (n=473) | 222 (46.9 %) | 160 (45.6 %) | 0.09844 | 0.75 |
| Low education level=TRUE (n=190) | 108 (56.8 %) | 274 (43.2 %) | 10.37 | 0.0013 |
| Living unsupervised=FALSE (n=142) | 85 (59.9 %) | 297 (43.5 %) | 11.93 | 0.00055 |
| Reports bullying=TRUE (n=478) | 241 (50.4 %) | 141 (40.8 %) | 7.159 | 0.0075 |
| Reports physical abuse=TRUE (n=204) | 121 (59.3 %) | 261 (42.1 %) | 17.61 | 2.70E-05 |
| Reports sexual abuse=TRUE (n=153) | 97 (63.4 %) | 285 (42.5 %) | 21.1 | 4.40E-06 |
| Used cannabis=TRUE (n=303) | 172 (56.8 %) | 210 (40.3 %) | 20.21 | 6.90E-06 |
| Used other drugs=TRUE (n=266) | 156 (58.6 %) | 226 (40.5 %) | 23.12 | 1.50E-06 |
| Hospitalizations over median=TRUE (n=485) | 276 (56.9 %) | 106 (31.3 %) | 51.72 | 6.40E-13 |
| Bipolar disorder (n=1439) | 665 (46.2 %) |  |  |  |
| Female=TRUE (n=906) | 441 (48.7 %) | 224 (42 %) | 5.704 | 0.017 |
| Working or studying=FALSE (n=1128) | 550 (48.8 %) | 115 (37 %) | 13.14 | 0.00029 |
| Has children=FALSE (n=594) | 270 (45.5 %) | 395 (46.7 %) | 0.1848 | 0.67 |
| Low education level=TRUE (n=282) | 153 (54.3 %) | 512 (44.3 %) | 8.729 | 0.0031 |
| Living unsupervised=FALSE (n=130) | 72 (55.4 %) | 593 (45.3 %) | 4.44 | 0.035 |
| Reports bullying=TRUE (n=805) | 422 (52.4 %) | 243 (38.3 %) | 27.78 | 1.40E-07 |
| Reports physical abuse=TRUE (n=398) | 244 (61.3 %) | 421 (40.4 %) | 49.59 | 1.90E-12 |
| Reports sexual abuse=TRUE (n=252) | 170 (67.5 %) | 495 (41.7 %) | 54.46 | 1.60E-13 |
| Used cannabis=TRUE (n=508) | 296 (58.3 %) | 369 (39.6 %) | 45.16 | 1.80E-11 |
| Used other drugs=TRUE (n=441) | 281 (63.7 %) | 384 (38.5 %) | 77.39 | 1.40E-18 |
| Hospitalizations over median=TRUE (n=540) | 339 (62.8 %) | 326 (36.3 %) | 94.36 | 2.60E-22 |
| Psychotic depression (n=464) | 208 (44.8 %) |  |  |  |
| Female=TRUE (n=288) | 134 (46.5 %) | 74 (42 %) | 0.7154 | 0.4 |
| Working or studying=FALSE (n=387) | 175 (45.2 %) | 33 (42.9 %) | 0.06515 | 0.8 |
| Has children=FALSE (n=254) | 122 (48 %) | 86 (41 %) | 2.052 | 0.15 |
| Low education level=TRUE (n=130) | 67 (51.5 %) | 141 (42.2 %) | 2.922 | 0.087 |
| Living unsupervised=FALSE (n=81) | 45 (55.6 %) | 163 (42.6 %) | 4.056 | 0.044 |
| Reports bullying=TRUE (n=251) | 128 (51 %) | 80 (37.6 %) | 7.877 | 0.005 |
| Reports physical abuse=TRUE (n=125) | 69 (55.2 %) | 139 (41 %) | 6.88 | 0.0087 |
| Reports sexual abuse=TRUE (n=86) | 55 (64 %) | 153 (40.5 %) | 14.68 | 0.00013 |
| Used cannabis=TRUE (n=102) | 63 (61.8 %) | 145 (40.1 %) | 14.3 | 0.00016 |
| Used other drugs=TRUE (n=95) | 64 (67.4 %) | 144 (39 %) | 23.41 | 1.30E-06 |
| Hospitalizations over median=TRUE (n=169) | 116 (68.6 %) | 92 (31.2 %) | 59.43 | 1.30E-14 |
| Other psychosis (n=899) | 217 (24.1 %) |  |  |  |
| Female=TRUE (n=438) | 116 (26.5 %) | 101 (21.9 %) | 2.324 | 0.13 |
| Working or studying=FALSE (n=675) | 177 (26.2 %) | 40 (17.9 %) | 5.978 | 0.014 |
| Has children=FALSE (n=621) | 160 (25.8 %) | 57 (20.5 %) | 2.623 | 0.11 |
| Low education level=TRUE (n=283) | 77 (27.2 %) | 140 (22.7 %) | 1.889 | 0.17 |
| Living unsupervised=FALSE (n=165) | 49 (29.7 %) | 168 (22.9 %) | 3.049 | 0.081 |
| Reports bullying=TRUE (n=501) | 150 (29.9 %) | 67 (16.8 %) | 20.1 | 7.40E-06 |
| Reports physical abuse=TRUE (n=205) | 89 (43.4 %) | 128 (18.4 %) | 52.53 | 4.20E-13 |
| Reports sexual abuse=TRUE (n=111) | 53 (47.7 %) | 164 (20.8 %) | 37.09 | 1.10E-09 |
| Used cannabis=TRUE (n=349) | 116 (33.2 %) | 101 (18.4 %) | 24.99 | 5.80E-07 |
| Used other drugs=TRUE (n=280) | 111 (39.6 %) | 106 (17.1 %) | 52.17 | 5.10E-13 |
| Hospitalizations over median=TRUE (n=150) | 59 (39.3 %) | 158 (21.1 %) | 21.72 | 3.20E-06 |
| **Description** | **Registry-based self-harm** | |  |  |
| All (n=7067) | 1364 (19.3 %) |  |  |  |
| Female=TRUE (n=3578) | 778 (21.7 %) | 586 (16.8 %) | 27.45 | 1.60E-07 |
| Working or studying=FALSE (n=5971) | 1226 (20.5 %) | 138 (12.6 %) | 36.99 | 1.20E-09 |
| Has children=FALSE (n=4536) | 846 (18.7 %) | 518 (20.5 %) | 3.322 | 0.068 |
| Low education level=TRUE (n=2108) | 529 (25.1 %) | 835 (16.8 %) | 64.22 | 1.10E-15 |
| Living unsupervised=FALSE (n=1678) | 430 (25.6 %) | 934 (17.3 %) | 55.98 | 7.30E-14 |
| Reports bullying=TRUE (n=3952) | 821 (20.8 %) | 543 (17.4 %) | 12.28 | 0.00046 |
| Reports physical abuse=TRUE (n=1795) | 415 (23.1 %) | 949 (18 %) | 22.2 | 2.50E-06 |
| Reports sexual abuse=TRUE (n=1041) | 285 (27.4 %) | 1079 (17.9 %) | 50.52 | 1.20E-12 |
| Used cannabis=TRUE (n=2514) | 670 (26.7 %) | 694 (15.2 %) | 134.6 | 4.00E-31 |
| Used other drugs=TRUE (n=2242) | 679 (30.3 %) | 685 (14.2 %) | 253.3 | 4.80E-57 |
| Hospitalizations over median=TRUE (n=3266) | 1029 (31.5 %) | 335 (8.8 %) | 579.3 | 5.30E-128 |
| Schizophrenia (n=3441) | 622 (18.1 %) | - | - |  |
| Female=TRUE (n=1429) | 299 (20.9 %) | 323 (16.1 %) | 13.06 | 3.00E-04 |
| Working or studying=FALSE (n=3081) | 572 (18.6 %) | 50 (13.9 %) | 4.45 | 0.035 |
| Has children=FALSE (n=2594) | 448 (17.3 %) | 174 (20.5 %) | 4.399 | 0.036 |
| Low education level=TRUE (n=1223) | 272 (22.2 %) | 350 (15.8 %) | 21.78 | 3.10E-06 |
| Living unsupervised=FALSE (n=1160) | 272 (23.4 %) | 350 (15.3 %) | 33.56 | 6.90E-09 |
| Reports bullying=TRUE (n=1917) | 373 (19.5 %) | 249 (16.3 %) | 5.368 | 0.021 |
| Reports physical abuse=TRUE (n=863) | 181 (21 %) | 441 (17.1 %) | 6.271 | 0.012 |
| Reports sexual abuse=TRUE (n=439) | 111 (25.3 %) | 511 (17 %) | 17.1 | 3.50E-05 |
| Used cannabis=TRUE (n=1252) | 315 (25.2 %) | 307 (14 %) | 65.94 | 4.70E-16 |
| Used other drugs=TRUE (n=1160) | 324 (27.9 %) | 298 (13.1 %) | 113.8 | 1.50E-26 |
| Hospitalizations over median=TRUE (n=1922) | 517 (26.9 %) | 105 (6.9 %) | 227.5 | 2.10E-51 |
| Schizoaffective disorder (n=824) | 189 (22.9 %) | - | - |  |
| Female=TRUE (n=517) | 120 (23.2 %) | 69 (22.5 %) | 0.02466 | 0.88 |
| Working or studying=FALSE (n=700) | 179 (25.6 %) | 10 (8.1 %) | 17.29 | 3.20E-05 |
| Has children=FALSE (n=473) | 110 (23.3 %) | 79 (22.5 %) | 0.02856 | 0.87 |
| Low education level=TRUE (n=190) | 61 (32.1 %) | 128 (20.2 %) | 11.08 | 0.00087 |
| Living unsupervised=FALSE (n=142) | 53 (37.3 %) | 136 (19.9 %) | 19.12 | 1.20E-05 |
| Reports bullying=TRUE (n=478) | 117 (24.5 %) | 72 (20.8 %) | 1.327 | 0.25 |
| Reports physical abuse=TRUE (n=204) | 49 (24 %) | 140 (22.6 %) | 0.1076 | 0.74 |
| Reports sexual abuse=TRUE (n=153) | 44 (28.8 %) | 145 (21.6 %) | 3.209 | 0.073 |
| Used cannabis=TRUE (n=303) | 91 (30 %) | 98 (18.8 %) | 13.02 | 0.00031 |
| Used other drugs=TRUE (n=266) | 86 (32.3 %) | 103 (18.5 %) | 18.83 | 1.40E-05 |
| Hospitalizations over median=TRUE (n=485) | 162 (33.4 %) | 27 (8 %) | 71.61 | 2.60E-17 |
| Bipolar disorder (n=1439) | 342 (23.8 %) | - | - |  |
| Female=TRUE (n=906) | 231 (25.5 %) | 111 (20.8 %) | 3.788 | 0.052 |
| Working or studying=FALSE (n=1128) | 299 (26.5 %) | 43 (13.8 %) | 20.94 | 4.70E-06 |
| Has children=FALSE (n=594) | 153 (25.8 %) | 189 (22.4 %) | 2.03 | 0.15 |
| Low education level=TRUE (n=282) | 99 (35.1 %) | 243 (21 %) | 24.12 | 9.00E-07 |
| Living unsupervised=FALSE (n=130) | 50 (38.5 %) | 292 (22.3 %) | 16.15 | 5.80E-05 |
| Reports bullying=TRUE (n=805) | 208 (25.8 %) | 134 (21.1 %) | 4.074 | 0.044 |
| Reports physical abuse=TRUE (n=398) | 111 (27.9 %) | 231 (22.2 %) | 4.852 | 0.028 |
| Reports sexual abuse=TRUE (n=252) | 76 (30.2 %) | 266 (22.4 %) | 6.469 | 0.011 |
| Used cannabis=TRUE (n=508) | 161 (31.7 %) | 181 (19.4 %) | 26.56 | 2.60E-07 |
| Used other drugs=TRUE (n=441) | 157 (35.6 %) | 185 (18.5 %) | 48.22 | 3.80E-12 |
| Hospitalizations over median=TRUE (n=540) | 226 (41.9 %) | 116 (12.9 %) | 154.4 | 1.80E-35 |
| Psychotic depression (n=464) | 111 (23.9 %) | - | - |  |
| Female=TRUE (n=288) | 77 (26.7 %) | 34 (19.3 %) | 2.908 | 0.088 |
| Working or studying=FALSE (n=387) | 92 (23.8 %) | 19 (24.7 %) | 0.00054 | 0.98 |
| Has children=FALSE (n=254) | 63 (24.8 %) | 48 (22.9 %) | 0.1442 | 0.7 |
| Low education level=TRUE (n=130) | 42 (32.3 %) | 69 (20.7 %) | 6.352 | 0.012 |
| Living unsupervised=FALSE (n=81) | 27 (33.3 %) | 84 (21.9 %) | 4.169 | 0.041 |
| Reports bullying=TRUE (n=251) | 61 (24.3 %) | 50 (23.5 %) | 0.00986 | 0.92 |
| Reports physical abuse=TRUE (n=125) | 39 (31.2 %) | 72 (21.2 %) | 4.447 | 0.035 |
| Reports sexual abuse=TRUE (n=86) | 32 (37.2 %) | 79 (20.9 %) | 9.364 | 0.0022 |
| Used cannabis=TRUE (n=102) | 44 (43.1 %) | 67 (18.5 %) | 25.19 | 5.20E-07 |
| Used other drugs=TRUE (n=95) | 44 (46.3 %) | 67 (18.2 %) | 31.39 | 2.10E-08 |
| Hospitalizations over median=TRUE (n=169) | 75 (44.4 %) | 36 (12.2 %) | 59.36 | 1.30E-14 |
| Other psychosis (n=899) | 100 (11.1 %) | - | - |  |
| Female=TRUE (n=438) | 51 (11.6 %) | 49 (10.6 %) | 0.1426 | 0.71 |
| Working or studying=FALSE (n=675) | 84 (12.4 %) | 16 (7.1 %) | 4.26 | 0.039 |
| Has children=FALSE (n=621) | 72 (11.6 %) | 28 (10.1 %) | 0.3093 | 0.58 |
| Low education level=TRUE (n=283) | 55 (19.4 %) | 45 (7.3 %) | 27.64 | 1.50E-07 |
| Living unsupervised=FALSE (n=165) | 28 (17 %) | 72 (9.8 %) | 6.281 | 0.012 |
| Reports bullying=TRUE (n=501) | 62 (12.4 %) | 38 (9.5 %) | 1.519 | 0.22 |
| Reports physical abuse=TRUE (n=205) | 35 (17.1 %) | 65 (9.4 %) | 8.745 | 0.0031 |
| Reports sexual abuse=TRUE (n=111) | 22 (19.8 %) | 78 (9.9 %) | 8.71 | 0.0032 |
| Used cannabis=TRUE (n=349) | 59 (16.9 %) | 41 (7.5 %) | 18.35 | 1.80E-05 |
| Used other drugs=TRUE (n=280) | 68 (24.3 %) | 32 (5.2 %) | 69.34 | 8.30E-17 |
| Hospitalizations over median=TRUE (n=150) | 49 (32.7 %) | 51 (6.8 %) | 81.93 | 1.40E-19 |
| **Description** | **Suicidal thoughts in past 12 months** | | |  |
| All (n=7067) | 2337 (33.1 %) |  |  |  |
| Female=TRUE (n=3578) | 1282 (35.8 %) | 1055 (30.2 %) | 24.71 | 6.70E-07 |
| Working or studying=FALSE (n=5971) | 1944 (32.6 %) | 393 (35.9 %) | 4.409 | 0.036 |
| Has children=FALSE (n=4536) | 1629 (35.9 %) | 708 (28 %) | 45.91 | 1.20E-11 |
| Low education level=TRUE (n=2108) | 717 (34 %) | 1620 (32.7 %) | 1.15 | 0.28 |
| Living unsupervised=FALSE (n=1678) | 489 (29.1 %) | 1848 (34.3 %) | 15.1 | 1.00E-04 |
| Reports bullying=TRUE (n=3952) | 1498 (37.9 %) | 839 (26.9 %) | 94.23 | 2.80E-22 |
| Reports physical abuse=TRUE (n=1795) | 751 (41.8 %) | 1586 (30.1 %) | 83.07 | 7.90E-20 |
| Reports sexual abuse=TRUE (n=1041) | 473 (45.4 %) | 1864 (30.9 %) | 83.72 | 5.70E-20 |
| Used cannabis=TRUE (n=2514) | 1035 (41.2 %) | 1302 (28.6 %) | 115.1 | 7.40E-27 |
| Used other drugs=TRUE (n=2242) | 948 (42.3 %) | 1389 (28.8 %) | 125.4 | 4.20E-29 |
| Hospitalizations over median=TRUE (n=3266) | 1141 (34.9 %) | 1196 (31.5 %) | 9.402 | 0.0022 |
| Schizophrenia (n=3441) | 919 (26.7 %) | - | - |  |
| Female=TRUE (n=1429) | 418 (29.3 %) | 501 (24.9 %) | 7.859 | 0.0051 |
| Working or studying=FALSE (n=3081) | 811 (26.3 %) | 108 (30 %) | 2.043 | 0.15 |
| Has children=FALSE (n=2594) | 755 (29.1 %) | 164 (19.4 %) | 30.47 | 3.40E-08 |
| Low education level=TRUE (n=1223) | 331 (27.1 %) | 588 (26.5 %) | 0.09701 | 0.76 |
| Living unsupervised=FALSE (n=1160) | 292 (25.2 %) | 627 (27.5 %) | 1.99 | 0.16 |
| Reports bullying=TRUE (n=1917) | 613 (32 %) | 306 (20.1 %) | 60.8 | 6.30E-15 |
| Reports physical abuse=TRUE (n=863) | 287 (33.3 %) | 632 (24.5 %) | 24.79 | 6.40E-07 |
| Reports sexual abuse=TRUE (n=439) | 163 (37.1 %) | 756 (25.2 %) | 27.32 | 1.70E-07 |
| Used cannabis=TRUE (n=1252) | 412 (32.9 %) | 507 (23.2 %) | 38.15 | 6.50E-10 |
| Used other drugs=TRUE (n=1160) | 393 (33.9 %) | 526 (23.1 %) | 45.43 | 1.60E-11 |
| Hospitalizations over median=TRUE (n=1922) | 558 (29 %) | 361 (23.8 %) | 11.76 | 0.00061 |
| Schizoaffective disorder (n=824) | 309 (37.5 %) | - | - |  |
| Female=TRUE (n=517) | 184 (35.6 %) | 125 (40.7 %) | 1.947 | 0.16 |
| Working or studying=FALSE (n=700) | 271 (38.7 %) | 38 (30.6 %) | 2.592 | 0.11 |
| Has children=FALSE (n=473) | 204 (43.1 %) | 105 (29.9 %) | 14.45 | 0.00014 |
| Low education level=TRUE (n=190) | 87 (45.8 %) | 222 (35 %) | 6.788 | 0.0092 |
| Living unsupervised=FALSE (n=142) | 59 (41.5 %) | 250 (36.7 %) | 1.001 | 0.32 |
| Reports bullying=TRUE (n=478) | 198 (41.4 %) | 111 (32.1 %) | 7.08 | 0.0078 |
| Reports physical abuse=TRUE (n=204) | 101 (49.5 %) | 208 (33.5 %) | 16.01 | 6.30E-05 |
| Reports sexual abuse=TRUE (n=153) | 70 (45.8 %) | 239 (35.6 %) | 5.035 | 0.025 |
| Used cannabis=TRUE (n=303) | 132 (43.6 %) | 177 (34 %) | 7.116 | 0.0076 |
| Used other drugs=TRUE (n=266) | 124 (46.6 %) | 185 (33.2 %) | 13.36 | 0.00026 |
| Hospitalizations over median=TRUE (n=485) | 185 (38.1 %) | 124 (36.6 %) | 0.1473 | 0.7 |
| Bipolar disorder (n=1439) | 597 (41.5 %) | - | - |  |
| Female=TRUE (n=906) | 394 (43.5 %) | 203 (38.1 %) | 3.814 | 0.051 |
| Working or studying=FALSE (n=1128) | 465 (41.2 %) | 132 (42.4 %) | 0.1035 | 0.75 |
| Has children=FALSE (n=594) | 293 (49.3 %) | 304 (36 %) | 25.06 | 5.60E-07 |
| Low education level=TRUE (n=282) | 129 (45.7 %) | 468 (40.4 %) | 2.405 | 0.12 |
| Living unsupervised=FALSE (n=130) | 60 (46.2 %) | 537 (41 %) | 1.079 | 0.3 |
| Reports bullying=TRUE (n=805) | 366 (45.5 %) | 231 (36.4 %) | 11.55 | 0.00068 |
| Reports physical abuse=TRUE (n=398) | 194 (48.7 %) | 403 (38.7 %) | 11.52 | 0.00069 |
| Reports sexual abuse=TRUE (n=252) | 137 (54.4 %) | 460 (38.8 %) | 20.23 | 6.90E-06 |
| Used cannabis=TRUE (n=508) | 265 (52.2 %) | 332 (35.7 %) | 36.2 | 1.80E-09 |
| Used other drugs=TRUE (n=441) | 240 (54.4 %) | 357 (35.8 %) | 43.06 | 5.30E-11 |
| Hospitalizations over median=TRUE (n=540) | 244 (45.2 %) | 353 (39.3 %) | 4.629 | 0.031 |
| Psychotic depression (n=464) | 234 (50.4 %) | - | - |  |
| Female=TRUE (n=288) | 160 (55.6 %) | 74 (42 %) | 7.445 | 0.0064 |
| Working or studying=FALSE (n=387) | 195 (50.4 %) | 39 (50.6 %) | 5.22E-31 | 1 |
| Has children=FALSE (n=254) | 150 (59.1 %) | 84 (40 %) | 15.94 | 6.50E-05 |
| Low education level=TRUE (n=130) | 73 (56.2 %) | 161 (48.2 %) | 2.059 | 0.15 |
| Living unsupervised=FALSE (n=81) | 35 (43.2 %) | 199 (52 %) | 1.712 | 0.19 |
| Reports bullying=TRUE (n=251) | 147 (58.6 %) | 87 (40.8 %) | 13.77 | 0.00021 |
| Reports physical abuse=TRUE (n=125) | 83 (66.4 %) | 151 (44.5 %) | 16.59 | 4.60E-05 |
| Reports sexual abuse=TRUE (n=86) | 50 (58.1 %) | 184 (48.7 %) | 2.145 | 0.14 |
| Used cannabis=TRUE (n=102) | 67 (65.7 %) | 167 (46.1 %) | 11.4 | 0.00073 |
| Used other drugs=TRUE (n=95) | 61 (64.2 %) | 173 (46.9 %) | 8.394 | 0.0038 |
| Hospitalizations over median=TRUE (n=169) | 99 (58.6 %) | 135 (45.8 %) | 6.558 | 0.01 |
| Other psychosis (n=899) | 278 (30.9 %) | - | - |  |
| Female=TRUE (n=438) | 126 (28.8 %) | 152 (33 %) | 1.667 | 0.2 |
| Working or studying=FALSE (n=675) | 202 (29.9 %) | 76 (33.9 %) | 1.081 | 0.3 |
| Has children=FALSE (n=621) | 227 (36.6 %) | 51 (18.3 %) | 28.96 | 7.40E-08 |
| Low education level=TRUE (n=283) | 97 (34.3 %) | 181 (29.4 %) | 1.95 | 0.16 |
| Living unsupervised=FALSE (n=165) | 43 (26.1 %) | 235 (32 %) | 1.967 | 0.16 |
| Reports bullying=TRUE (n=501) | 174 (34.7 %) | 104 (26.1 %) | 7.282 | 0.007 |
| Reports physical abuse=TRUE (n=205) | 86 (42 %) | 192 (27.7 %) | 14.46 | 0.00014 |
| Reports sexual abuse=TRUE (n=111) | 53 (47.7 %) | 225 (28.6 %) | 15.89 | 6.70E-05 |
| Used cannabis=TRUE (n=349) | 159 (45.6 %) | 119 (21.6 %) | 56.09 | 6.90E-14 |
| Used other drugs=TRUE (n=280) | 130 (46.4 %) | 148 (23.9 %) | 44.72 | 2.30E-11 |
| Hospitalizations over median=TRUE (n=150) | 55 (36.7 %) | 223 (29.8 %) | 2.467 | 0.12 |
| **Description** | **Violent self-harm episode (participants)** | | |  |
| All (n=7067) | 282 (4 %) |  |  |  |
| Female=TRUE (n=3578) | 156 (4.4 %) | 126 (3.6 %) | 2.392 | 0.12 |
| Working or studying=FALSE (n=5971) | 246 (4.1 %) | 36 (3.3 %) | 1.475 | 0.22 |
| Has children=FALSE (n=4536) | 206 (4.5 %) | 76 (3 %) | 9.642 | 0.0019 |
| Low education level=TRUE (n=2108) | 130 (6.2 %) | 152 (3.1 %) | 36.34 | 1.70E-09 |
| Living unsupervised=FALSE (n=1678) | 114 (6.8 %) | 168 (3.1 %) | 44.19 | 3.00E-11 |
| Reports bullying=TRUE (n=3952) | 180 (4.6 %) | 102 (3.3 %) | 7.121 | 0.0076 |
| Reports physical abuse=TRUE (n=1795) | 84 (4.7 %) | 198 (3.8 %) | 2.748 | 0.097 |
| Reports sexual abuse=TRUE (n=1041) | 60 (5.8 %) | 222 (3.7 %) | 9.485 | 0.0021 |
| Used cannabis=TRUE (n=2514) | 144 (5.7 %) | 138 (3 %) | 30.05 | 4.20E-08 |
| Used other drugs=TRUE (n=2242) | 139 (6.2 %) | 143 (3 %) | 41 | 1.50E-10 |
| Hospitalizations over median=TRUE (n=3266) | 213 (6.5 %) | 69 (1.8 %) | 100.3 | 1.30E-23 |
| Schizophrenia (n=3441) | 135 (3.9 %) | - | - |  |
| Female=TRUE (n=1429) | 62 (4.3 %) | 73 (3.6 %) | 0.9384 | 0.33 |
| Working or studying=FALSE (n=3081) | 118 (3.8 %) | 17 (4.7 %) | 0.4647 | 0.5 |
| Has children=FALSE (n=2594) | 110 (4.2 %) | 25 (3 %) | 2.483 | 0.12 |
| Low education level=TRUE (n=1223) | 71 (5.8 %) | 64 (2.9 %) | 17.06 | 3.60E-05 |
| Living unsupervised=FALSE (n=1160) | 73 (6.3 %) | 62 (2.7 %) | 25.13 | 5.40E-07 |
| Reports bullying=TRUE (n=1917) | 83 (4.3 %) | 52 (3.4 %) | 1.661 | 0.2 |
| Reports physical abuse=TRUE (n=863) | 37 (4.3 %) | 98 (3.8 %) | 0.2864 | 0.59 |
| Reports sexual abuse=TRUE (n=439) | 22 (5 %) | 113 (3.8 %) | 1.267 | 0.26 |
| Used cannabis=TRUE (n=1252) | 70 (5.6 %) | 65 (3 %) | 13.84 | 2.00E-04 |
| Used other drugs=TRUE (n=1160) | 69 (5.9 %) | 66 (2.9 %) | 18.24 | 2.00E-05 |
| Hospitalizations over median=TRUE (n=1922) | 104 (5.4 %) | 31 (2 %) | 24.68 | 6.80E-07 |
| Schizoaffective disorder (n=824) | 39 (4.7 %) | - | - |  |
| Female=TRUE (n=517) | 22 (4.3 %) | 17 (5.5 %) | 0.4467 | 0.5 |
| Working or studying=FALSE (n=700) | 38 (5.4 %) | 1 (0.8 %) | 4.019 | 0.045 |
| Has children=FALSE (n=473) | 26 (5.5 %) | 13 (3.7 %) | 1.067 | 0.3 |
| Low education level=TRUE (n=190) | 14 (7.4 %) | 25 (3.9 %) | 3.082 | 0.079 |
| Living unsupervised=FALSE (n=142) | 13 (9.2 %) | 26 (3.8 %) | 6.302 | 0.012 |
| Reports bullying=TRUE (n=478) | 23 (4.8 %) | 16 (4.6 %) | 1.20E-29 | 1 |
| Reports physical abuse=TRUE (n=204) | 11 (5.4 %) | 28 (4.5 %) | 0.1031 | 0.75 |
| Reports sexual abuse=TRUE (n=153) | 6 (3.9 %) | 33 (4.9 %) | 0.09787 | 0.75 |
| Used cannabis=TRUE (n=303) | 16 (5.3 %) | 23 (4.4 %) | 0.1555 | 0.69 |
| Used other drugs=TRUE (n=266) | 17 (6.4 %) | 22 (3.9 %) | 1.882 | 0.17 |
| Hospitalizations over median=TRUE (n=485) | 34 (7 %) | 5 (1.5 %) | 12.36 | 0.00044 |
| Bipolar disorder (n=1439) | 54 (3.8 %) | - | - |  |
| Female=TRUE (n=906) | 41 (4.5 %) | 13 (2.4 %) | 3.487 | 0.062 |
| Working or studying=FALSE (n=1128) | 48 (4.3 %) | 6 (1.9 %) | 3.036 | 0.081 |
| Has children=FALSE (n=594) | 30 (5.1 %) | 24 (2.8 %) | 4.126 | 0.042 |
| Low education level=TRUE (n=282) | 21 (7.4 %) | 33 (2.9 %) | 12.01 | 0.00053 |
| Living unsupervised=FALSE (n=130) | 13 (10 %) | 41 (3.1 %) | 13.6 | 0.00023 |
| Reports bullying=TRUE (n=805) | 39 (4.8 %) | 15 (2.4 %) | 5.367 | 0.021 |
| Reports physical abuse=TRUE (n=398) | 19 (4.8 %) | 35 (3.4 %) | 1.222 | 0.27 |
| Reports sexual abuse=TRUE (n=252) | 16 (6.3 %) | 38 (3.2 %) | 4.865 | 0.027 |
| Used cannabis=TRUE (n=508) | 29 (5.7 %) | 25 (2.7 %) | 7.502 | 0.0062 |
| Used other drugs=TRUE (n=441) | 27 (6.1 %) | 27 (2.7 %) | 8.964 | 0.0028 |
| Hospitalizations over median=TRUE (n=540) | 43 (8 %) | 11 (1.2 %) | 40.58 | 1.90E-10 |
| Psychotic depression (n=464) | 33 (7.1 %) | - | - |  |
| Female=TRUE (n=288) | 22 (7.6 %) | 11 (6.2 %) | 0.1434 | 0.7 |
| Working or studying=FALSE (n=387) | 27 (7 %) | 6 (7.8 %) | 0.00013 | 0.99 |
| Has children=FALSE (n=254) | 24 (9.4 %) | 9 (4.3 %) | 3.89 | 0.049 |
| Low education level=TRUE (n=130) | 14 (10.8 %) | 19 (5.7 %) | 2.928 | 0.087 |
| Living unsupervised=FALSE (n=81) | 9 (11.1 %) | 24 (6.3 %) | 1.699 | 0.19 |
| Reports bullying=TRUE (n=251) | 23 (9.2 %) | 10 (4.7 %) | 2.839 | 0.092 |
| Reports physical abuse=TRUE (n=125) | 14 (11.2 %) | 19 (5.6 %) | 3.522 | 0.061 |
| Reports sexual abuse=TRUE (n=86) | 11 (12.8 %) | 22 (5.8 %) | 4.152 | 0.042 |
| Used cannabis=TRUE (n=102) | 15 (14.7 %) | 18 (5 %) | 9.987 | 0.0016 |
| Used other drugs=TRUE (n=95) | 11 (11.6 %) | 22 (6 %) | 2.808 | 0.094 |
| Hospitalizations over median=TRUE (n=169) | 24 (14.2 %) | 9 (3.1 %) | 18.57 | 1.60E-05 |
| Other psychosis (n=899) | 21 (2.3 %) | - | - |  |
| Female=TRUE (n=438) | 9 (2.1 %) | 12 (2.6 %) | 0.1044 | 0.75 |
| Working or studying=FALSE (n=675) | 15 (2.2 %) | 6 (2.7 %) | 0.01865 | 0.89 |
| Has children=FALSE (n=621) | 16 (2.6 %) | 5 (1.8 %) | 0.2255 | 0.63 |
| Low education level=TRUE (n=283) | 10 (3.5 %) | 11 (1.8 %) | 1.887 | 0.17 |
| Living unsupervised=FALSE (n=165) | 6 (3.6 %) | 15 (2 %) | 0.8812 | 0.35 |
| Reports bullying=TRUE (n=501) | 12 (2.4 %) | 9 (2.3 %) | 1.46E-30 | 1 |
| Reports physical abuse=TRUE (n=205) | 3 (1.5 %) | 18 (2.6 %) | 0.46 | 0.5 |
| Reports sexual abuse=TRUE (n=111) | 5 (4.5 %) | 16 (2 %) | 1.639 | 0.2 |
| Used cannabis=TRUE (n=349) | 14 (4 %) | 7 (1.3 %) | 5.871 | 0.015 |
| Used other drugs=TRUE (n=280) | 15 (5.4 %) | 6 (1 %) | 14.4 | 0.00015 |
| Hospitalizations over median=TRUE (n=150) | 8 (5.3 %) | 13 (1.7 %) | 5.601 | 0.018 |
| **Description** | **Reports multiple suicide attempts** | | |  |
| All (n=7067) | 1442 (20.4 %) |  |  |  |
| Female=TRUE (n=3578) | 880 (24.6 %) | 562 (16.1 %) | 77.82 | 1.10E-18 |
| Working or studying=FALSE (n=5971) | 1280 (21.4 %) | 162 (14.8 %) | 24.85 | 6.20E-07 |
| Has children=FALSE (n=4536) | 876 (19.3 %) | 566 (22.4 %) | 9.121 | 0.0025 |
| Low education level=TRUE (n=2108) | 513 (24.3 %) | 929 (18.7 %) | 28.24 | 1.10E-07 |
| Living unsupervised=FALSE (n=1678) | 427 (25.4 %) | 1015 (18.8 %) | 34.04 | 5.40E-09 |
| Reports bullying=TRUE (n=3952) | 954 (24.1 %) | 488 (15.7 %) | 76.49 | 2.20E-18 |
| Reports physical abuse=TRUE (n=1795) | 539 (30 %) | 903 (17.1 %) | 136.4 | 1.60E-31 |
| Reports sexual abuse=TRUE (n=1041) | 398 (38.2 %) | 1044 (17.3 %) | 237.6 | 1.30E-53 |
| Used cannabis=TRUE (n=2514) | 666 (26.5 %) | 776 (17 %) | 88.44 | 5.20E-21 |
| Used other drugs=TRUE (n=2242) | 688 (30.7 %) | 754 (15.6 %) | 212.8 | 3.30E-48 |
| Hospitalizations over median=TRUE (n=3266) | 1004 (30.7 %) | 438 (11.5 %) | 398.3 | 1.30E-88 |
| Schizophrenia (n=3441) | 639 (18.6 %) | - | - |  |
| Female=TRUE (n=1429) | 345 (24.1 %) | 294 (14.6 %) | 49.56 | 1.90E-12 |
| Working or studying=FALSE (n=3081) | 591 (19.2 %) | 48 (13.3 %) | 6.91 | 0.0086 |
| Has children=FALSE (n=2594) | 461 (17.8 %) | 178 (21 %) | 4.23 | 0.04 |
| Low education level=TRUE (n=1223) | 262 (21.4 %) | 377 (17 %) | 9.919 | 0.0016 |
| Living unsupervised=FALSE (n=1160) | 261 (22.5 %) | 378 (16.6 %) | 17.48 | 2.90E-05 |
| Reports bullying=TRUE (n=1917) | 415 (21.6 %) | 224 (14.7 %) | 26.66 | 2.40E-07 |
| Reports physical abuse=TRUE (n=863) | 222 (25.7 %) | 417 (16.2 %) | 38.36 | 5.90E-10 |
| Reports sexual abuse=TRUE (n=439) | 159 (36.2 %) | 480 (16 %) | 102.3 | 4.70E-24 |
| Used cannabis=TRUE (n=1252) | 284 (22.7 %) | 355 (16.2 %) | 21.6 | 3.40E-06 |
| Used other drugs=TRUE (n=1160) | 299 (25.8 %) | 340 (14.9 %) | 59.37 | 1.30E-14 |
| Hospitalizations over median=TRUE (n=1922) | 495 (25.8 %) | 144 (9.5 %) | 147.5 | 6.00E-34 |
| Schizoaffective disorder (n=824) | 200 (24.3 %) | - | - |  |
| Female=TRUE (n=517) | 130 (25.1 %) | 70 (22.8 %) | 0.4552 | 0.5 |
| Working or studying=FALSE (n=700) | 185 (26.4 %) | 15 (12.1 %) | 11 | 0.00091 |
| Has children=FALSE (n=473) | 116 (24.5 %) | 84 (23.9 %) | 0.01301 | 0.91 |
| Low education level=TRUE (n=190) | 62 (32.6 %) | 138 (21.8 %) | 8.807 | 0.003 |
| Living unsupervised=FALSE (n=142) | 57 (40.1 %) | 143 (21 %) | 22.47 | 2.10E-06 |
| Reports bullying=TRUE (n=478) | 133 (27.8 %) | 67 (19.4 %) | 7.362 | 0.0067 |
| Reports physical abuse=TRUE (n=204) | 67 (32.8 %) | 133 (21.5 %) | 10.23 | 0.0014 |
| Reports sexual abuse=TRUE (n=153) | 57 (37.3 %) | 143 (21.3 %) | 16.37 | 5.20E-05 |
| Used cannabis=TRUE (n=303) | 95 (31.4 %) | 105 (20.2 %) | 12.47 | 0.00041 |
| Used other drugs=TRUE (n=266) | 95 (35.7 %) | 105 (18.8 %) | 27.07 | 2.00E-07 |
| Hospitalizations over median=TRUE (n=485) | 159 (32.8 %) | 41 (12.1 %) | 45.35 | 1.70E-11 |
| Bipolar disorder (n=1439) | 392 (27.2 %) | - | - |  |
| Female=TRUE (n=906) | 279 (30.8 %) | 113 (21.2 %) | 15.1 | 1.00E-04 |
| Working or studying=FALSE (n=1128) | 326 (28.9 %) | 66 (21.2 %) | 6.87 | 0.0088 |
| Has children=FALSE (n=594) | 157 (26.4 %) | 235 (27.8 %) | 0.269 | 0.6 |
| Low education level=TRUE (n=282) | 100 (35.5 %) | 292 (25.2 %) | 11.45 | 0.00072 |
| Living unsupervised=FALSE (n=130) | 49 (37.7 %) | 343 (26.2 %) | 7.307 | 0.0069 |
| Reports bullying=TRUE (n=805) | 259 (32.2 %) | 133 (21 %) | 21.87 | 2.90E-06 |
| Reports physical abuse=TRUE (n=398) | 154 (38.7 %) | 238 (22.9 %) | 35.61 | 2.40E-09 |
| Reports sexual abuse=TRUE (n=252) | 117 (46.4 %) | 275 (23.2 %) | 55.58 | 9.00E-14 |
| Used cannabis=TRUE (n=508) | 190 (37.4 %) | 202 (21.7 %) | 40.11 | 2.40E-10 |
| Used other drugs=TRUE (n=441) | 184 (41.7 %) | 208 (20.8 %) | 66.24 | 4.00E-16 |
| Hospitalizations over median=TRUE (n=540) | 233 (43.1 %) | 159 (17.7 %) | 109.1 | 1.60E-25 |
| Psychotic depression (n=464) | 118 (25.4 %) | - | - |  |
| Female=TRUE (n=288) | 76 (26.4 %) | 42 (23.9 %) | 0.2463 | 0.62 |
| Working or studying=FALSE (n=387) | 99 (25.6 %) | 19 (24.7 %) | 0.00055 | 0.98 |
| Has children=FALSE (n=254) | 73 (28.7 %) | 45 (21.4 %) | 2.867 | 0.09 |
| Low education level=TRUE (n=130) | 45 (34.6 %) | 73 (21.9 %) | 7.374 | 0.0066 |
| Living unsupervised=FALSE (n=81) | 29 (35.8 %) | 89 (23.2 %) | 4.923 | 0.026 |
| Reports bullying=TRUE (n=251) | 78 (31.1 %) | 40 (18.8 %) | 8.55 | 0.0035 |
| Reports physical abuse=TRUE (n=125) | 51 (40.8 %) | 67 (19.8 %) | 20.22 | 6.90E-06 |
| Reports sexual abuse=TRUE (n=86) | 38 (44.2 %) | 80 (21.2 %) | 18.39 | 1.80E-05 |
| Used cannabis=TRUE (n=102) | 39 (38.2 %) | 79 (21.8 %) | 10.45 | 0.0012 |
| Used other drugs=TRUE (n=95) | 46 (48.4 %) | 72 (19.5 %) | 31.79 | 1.70E-08 |
| Hospitalizations over median=TRUE (n=169) | 79 (46.7 %) | 39 (13.2 %) | 61.93 | 3.60E-15 |
| Other psychosis (n=899) | 93 (10.3 %) | - | - |  |
| Female=TRUE (n=438) | 50 (11.4 %) | 43 (9.3 %) | 0.8426 | 0.36 |
| Working or studying=FALSE (n=675) | 79 (11.7 %) | 14 (6.2 %) | 4.822 | 0.028 |
| Has children=FALSE (n=621) | 69 (11.1 %) | 24 (8.6 %) | 1.018 | 0.31 |
| Low education level=TRUE (n=283) | 44 (15.5 %) | 49 (8 %) | 11.25 | 8.00E-04 |
| Living unsupervised=FALSE (n=165) | 31 (18.8 %) | 62 (8.4 %) | 14.44 | 0.00014 |
| Reports bullying=TRUE (n=501) | 69 (13.8 %) | 24 (6 %) | 13.51 | 0.00024 |
| Reports physical abuse=TRUE (n=205) | 45 (22 %) | 48 (6.9 %) | 36.97 | 1.20E-09 |
| Reports sexual abuse=TRUE (n=111) | 27 (24.3 %) | 66 (8.4 %) | 24.99 | 5.80E-07 |
| Used cannabis=TRUE (n=349) | 58 (16.6 %) | 35 (6.4 %) | 23.12 | 1.50E-06 |
| Used other drugs=TRUE (n=280) | 64 (22.9 %) | 29 (4.7 %) | 66.7 | 3.20E-16 |
| Hospitalizations over median=TRUE (n=150) | 38 (25.3 %) | 55 (7.3 %) | 41.69 | 1.10E-10 |
| **Description** | **Multiple self-harm episodes in registry (participants)** | | | |
| All (n=7067) | 512 (7.2 %) |  |  |  |
| Female=TRUE (n=3578) | 332 (9.3 %) | 180 (5.2 %) | 44.01 | 3.30E-11 |
| Working or studying=FALSE (n=5971) | 465 (7.8 %) | 47 (4.3 %) | 16.36 | 5.20E-05 |
| Has children=FALSE (n=4536) | 331 (7.3 %) | 181 (7.2 %) | 0.03201 | 0.86 |
| Low education level=TRUE (n=2108) | 221 (10.5 %) | 291 (5.9 %) | 46.21 | 1.10E-11 |
| Living unsupervised=FALSE (n=1678) | 161 (9.6 %) | 351 (6.5 %) | 17.62 | 2.70E-05 |
| Reports bullying=TRUE (n=3952) | 327 (8.3 %) | 185 (5.9 %) | 13.79 | 2.00E-04 |
| Reports physical abuse=TRUE (n=1795) | 163 (9.1 %) | 349 (6.6 %) | 11.7 | 0.00062 |
| Reports sexual abuse=TRUE (n=1041) | 131 (12.6 %) | 381 (6.3 %) | 50.86 | 9.90E-13 |
| Used cannabis=TRUE (n=2514) | 261 (10.4 %) | 251 (5.5 %) | 56.42 | 5.90E-14 |
| Used other drugs=TRUE (n=2242) | 278 (12.4 %) | 234 (4.8 %) | 128.7 | 7.80E-30 |
| Hospitalizations over median=TRUE (n=3266) | 434 (13.3 %) | 78 (2.1 %) | 328.4 | 2.20E-73 |
| Schizophrenia (n=3441) | 205 (6 %) | - | - |  |
| Female=TRUE (n=1429) | 112 (7.8 %) | 93 (4.6 %) | 14.85 | 0.00012 |
| Working or studying=FALSE (n=3081) | 193 (6.3 %) | 12 (3.3 %) | 4.433 | 0.035 |
| Has children=FALSE (n=2594) | 149 (5.7 %) | 56 (6.6 %) | 0.7099 | 0.4 |
| Low education level=TRUE (n=1223) | 104 (8.5 %) | 101 (4.6 %) | 21.25 | 4.00E-06 |
| Living unsupervised=FALSE (n=1160) | 93 (8 %) | 112 (4.9 %) | 12.7 | 0.00037 |
| Reports bullying=TRUE (n=1917) | 127 (6.6 %) | 78 (5.1 %) | 3.177 | 0.075 |
| Reports physical abuse=TRUE (n=863) | 62 (7.2 %) | 143 (5.5 %) | 2.808 | 0.094 |
| Reports sexual abuse=TRUE (n=439) | 46 (10.5 %) | 159 (5.3 %) | 17.44 | 3.00E-05 |
| Used cannabis=TRUE (n=1252) | 102 (8.1 %) | 103 (4.7 %) | 16.23 | 5.60E-05 |
| Used other drugs=TRUE (n=1160) | 106 (9.1 %) | 99 (4.3 %) | 30.74 | 2.90E-08 |
| Hospitalizations over median=TRUE (n=1922) | 187 (9.7 %) | 18 (1.2 %) | 109 | 1.60E-25 |
| Schizoaffective disorder (n=824) | 71 (8.6 %) | - | - |  |
| Female=TRUE (n=517) | 55 (10.6 %) | 16 (5.2 %) | 6.531 | 0.011 |
| Working or studying=FALSE (n=700) | 68 (9.7 %) | 3 (2.4 %) | 6.223 | 0.013 |
| Has children=FALSE (n=473) | 48 (10.1 %) | 23 (6.6 %) | 2.867 | 0.09 |
| Low education level=TRUE (n=190) | 27 (14.2 %) | 44 (6.9 %) | 8.912 | 0.0028 |
| Living unsupervised=FALSE (n=142) | 20 (14.1 %) | 51 (7.5 %) | 5.703 | 0.017 |
| Reports bullying=TRUE (n=478) | 44 (9.2 %) | 27 (7.8 %) | 0.3385 | 0.56 |
| Reports physical abuse=TRUE (n=204) | 18 (8.8 %) | 53 (8.5 %) | 3.22E-30 | 1 |
| Reports sexual abuse=TRUE (n=153) | 20 (13.1 %) | 51 (7.6 %) | 4.067 | 0.044 |
| Used cannabis=TRUE (n=303) | 40 (13.2 %) | 31 (6 %) | 11.89 | 0.00056 |
| Used other drugs=TRUE (n=266) | 39 (14.7 %) | 32 (5.7 %) | 17.11 | 3.50E-05 |
| Hospitalizations over median=TRUE (n=485) | 70 (14.4 %) | 1 (0.3 %) | 48.87 | 2.70E-12 |
| Bipolar disorder (n=1439) | 152 (10.6 %) | - | - |  |
| Female=TRUE (n=906) | 108 (11.9 %) | 44 (8.3 %) | 4.392 | 0.036 |
| Working or studying=FALSE (n=1128) | 135 (12 %) | 17 (5.5 %) | 10.23 | 0.0014 |
| Has children=FALSE (n=594) | 75 (12.6 %) | 77 (9.1 %) | 4.194 | 0.041 |
| Low education level=TRUE (n=282) | 52 (18.4 %) | 100 (8.6 %) | 22.01 | 2.70E-06 |
| Living unsupervised=FALSE (n=130) | 25 (19.2 %) | 127 (9.7 %) | 10.38 | 0.0013 |
| Reports bullying=TRUE (n=805) | 102 (12.7 %) | 50 (7.9 %) | 8.095 | 0.0044 |
| Reports physical abuse=TRUE (n=398) | 52 (13.1 %) | 100 (9.6 %) | 3.29 | 0.07 |
| Reports sexual abuse=TRUE (n=252) | 37 (14.7 %) | 115 (9.7 %) | 4.972 | 0.026 |
| Used cannabis=TRUE (n=508) | 74 (14.6 %) | 78 (8.4 %) | 12.68 | 0.00037 |
| Used other drugs=TRUE (n=441) | 82 (18.6 %) | 70 (7 %) | 42.2 | 8.30E-11 |
| Hospitalizations over median=TRUE (n=540) | 115 (21.3 %) | 37 (4.1 %) | 103.6 | 2.50E-24 |
| Psychotic depression (n=464) | 48 (10.3 %) | - | - |  |
| Female=TRUE (n=288) | 39 (13.5 %) | 9 (5.1 %) | 7.482 | 0.0062 |
| Working or studying=FALSE (n=387) | 40 (10.3 %) | 8 (10.4 %) | 6.70E-30 | 1 |
| Has children=FALSE (n=254) | 32 (12.6 %) | 16 (7.6 %) | 2.56 | 0.11 |
| Low education level=TRUE (n=130) | 18 (13.8 %) | 30 (9 %) | 1.892 | 0.17 |
| Living unsupervised=FALSE (n=81) | 11 (13.6 %) | 37 (9.7 %) | 0.7253 | 0.39 |
| Reports bullying=TRUE (n=251) | 32 (12.7 %) | 16 (7.5 %) | 2.866 | 0.09 |
| Reports physical abuse=TRUE (n=125) | 20 (16 %) | 28 (8.3 %) | 5.095 | 0.024 |
| Reports sexual abuse=TRUE (n=86) | 18 (20.9 %) | 30 (7.9 %) | 11.39 | 0.00074 |
| Used cannabis=TRUE (n=102) | 23 (22.5 %) | 25 (6.9 %) | 19.34 | 1.10E-05 |
| Used other drugs=TRUE (n=95) | 24 (25.3 %) | 24 (6.5 %) | 26.68 | 2.40E-07 |
| Hospitalizations over median=TRUE (n=169) | 37 (21.9 %) | 11 (3.7 %) | 36.29 | 1.70E-09 |
| Other psychosis (n=899) | 36 (4 %) | - | - |  |
| Female=TRUE (n=438) | 18 (4.1 %) | 18 (3.9 %) | 2.11E-30 | 1 |
| Working or studying=FALSE (n=675) | 29 (4.3 %) | 7 (3.1 %) | 0.3342 | 0.56 |
| Has children=FALSE (n=621) | 27 (4.3 %) | 9 (3.2 %) | 0.361 | 0.55 |
| Low education level=TRUE (n=283) | 20 (7.1 %) | 16 (2.6 %) | 8.949 | 0.0028 |
| Living unsupervised=FALSE (n=165) | 12 (7.3 %) | 24 (3.3 %) | 4.622 | 0.032 |
| Reports bullying=TRUE (n=501) | 22 (4.4 %) | 14 (3.5 %) | 0.2424 | 0.62 |
| Reports physical abuse=TRUE (n=205) | 11 (5.4 %) | 25 (3.6 %) | 0.8627 | 0.35 |
| Reports sexual abuse=TRUE (n=111) | 10 (9 %) | 26 (3.3 %) | 6.832 | 0.009 |
| Used cannabis=TRUE (n=349) | 22 (6.3 %) | 14 (2.5 %) | 6.898 | 0.0086 |
| Used other drugs=TRUE (n=280) | 27 (9.6 %) | 9 (1.5 %) | 31.53 | 2.00E-08 |
| Hospitalizations over median=TRUE (n=150) | 25 (16.7 %) | 11 (1.5 %) | 71.19 | 3.20E-17 |
| **Description** | **Violent self-harm** | |  |  |
| **Self-harm episodes** | **Factor present** | **Factor absent** | **statistic** | **p-value** |
| All (n=3365) | 659 (19.6 %) |  |  |  |
| Earlier self-harm=TRUE (n=1999) | 458 (22.9 %) | 201 (14.7 %) | 34.1 | 5.20E-09 |
| Earlier violent self-harm=TRUE (n=696) | 377 (54.2 %) | 282 (10.6 %) | 663.6 | 2.40E-146 |
| First self-harm=TRUE (n=1366) | 201 (14.7 %) | 458 (22.9 %) | 34.1 | 5.20E-09 |
| Schizophrenia (n=1331) | 299 (22.5 %) |  |  |  |
| Earlier self-harm=TRUE (n=709) | 199 (28.1 %) | 100 (16.1 %) | 26.66 | 2.40E-07 |
| Earlier violent self-harm=TRUE (n=262) | 164 (62.6 %) | 135 (12.6 %) | 298.8 | 6.10E-67 |
| First self-harm=TRUE (n=622) | 100 (16.1 %) | 199 (28.1 %) | 26.66 | 2.40E-07 |
| Schizoaffective disorder (n=406) | 62 (15.3 %) |  |  |  |
| Earlier self-harm=TRUE (n=216) | 34 (15.7 %) | 28 (14.7 %) | 0.02026 | 0.89 |
| Earlier violent self-harm=TRUE (n=40) | 23 (57.5 %) | 39 (10.7 %) | 57.59 | 3.20E-14 |
| First self-harm=TRUE (n=190) | 28 (14.7 %) | 34 (15.7 %) | 0.02026 | 0.89 |
| Bipolar disorder (n=1068) | 123 (11.5 %) |  |  |  |
| Earlier self-harm=TRUE (n=726) | 93 (12.8 %) | 30 (8.8 %) | 3.334 | 0.068 |
| Earlier violent self-harm=TRUE (n=199) | 69 (34.7 %) | 54 (6.2 %) | 125.9 | 3.20E-29 |
| First self-harm=TRUE (n=342) | 30 (8.8 %) | 93 (12.8 %) | 3.334 | 0.068 |
| Psychotic depression (n=373) | 125 (33.5 %) |  |  |  |
| Earlier self-harm=TRUE (n=261) | 100 (38.3 %) | 25 (22.3 %) | 8.293 | 0.004 |
| Earlier violent self-harm=TRUE (n=153) | 92 (60.1 %) | 33 (15 %) | 80.48 | 2.90E-19 |
| First self-harm=TRUE (n=112) | 25 (22.3 %) | 100 (38.3 %) | 8.293 | 0.004 |
| Other psychosis (n=187) | 50 (26.7 %) | - |  |  |
| Earlier self-harm=TRUE (n=87) | 32 (36.8 %) | 18 (18 %) | 7.447 | 0.0064 |
| Earlier violent self-harm=TRUE (n=42) | 29 (69 %) | 21 (14.5 %) | 46.75 | 8.10E-12 |
| First self-harm=TRUE (n=100) | 18 (18 %) | 32 (36.8 %) | 7.447 | 0.0064 |
| **Description** | **Followed by self-harm episode** | |  |  |
| **Self-harm episodes** | **Factor present** | **Factor absent** | **statistic** | **p-value** |
| All (n=3365) | 2000 (59.4 %) | - | - |  |
| Earlier self-harm=TRUE (n=1999) | 1486 (74.3 %) | 514 (37.6 %) | 452 | 2.60E-100 |
| Earlier violent self-harm=TRUE (n=696) | 574 (82.5 %) | 1426 (53.4 %) | 191.9 | 1.20E-43 |
| First self-harm=TRUE (n=1366) | 514 (37.6 %) | 1486 (74.3 %) | 452 | 2.60E-100 |
| Schizophrenia (n=1331) | 709 (53.3 %) | - | - |  |
| Earlier self-harm=TRUE (n=709) | 504 (71.1 %) | 205 (33 %) | 192 | 1.20E-43 |
| Earlier violent self-harm=TRUE (n=262) | 213 (81.3 %) | 496 (46.4 %) | 101.6 | 6.90E-24 |
| First self-harm=TRUE (n=622) | 205 (33 %) | 504 (71.1 %) | 192 | 1.20E-43 |
| Schizoaffective disorder (n=406) | 216 (53.2 %) | - | - |  |
| Earlier self-harm=TRUE (n=216) | 144 (66.7 %) | 72 (37.9 %) | 32.46 | 1.20E-08 |
| Earlier violent self-harm=TRUE (n=40) | 21 (52.5 %) | 195 (53.3 %) | 0 | 1 |
| First self-harm=TRUE (n=190) | 72 (37.9 %) | 144 (66.7 %) | 32.46 | 1.20E-08 |
| Bipolar disorder (n=1068) | 726 (68 %) | - | - |  |
| Earlier self-harm=TRUE (n=726) | 574 (79.1 %) | 152 (44.4 %) | 126.4 | 2.50E-29 |
| Earlier violent self-harm=TRUE (n=199) | 170 (85.4 %) | 556 (64 %) | 33.23 | 8.20E-09 |
| First self-harm=TRUE (n=342) | 152 (44.4 %) | 574 (79.1 %) | 126.4 | 2.50E-29 |
| Psychotic depression (n=373) | 262 (70.2 %) | - | - |  |
| Earlier self-harm=TRUE (n=261) | 213 (81.6 %) | 49 (43.8 %) | 51.94 | 5.70E-13 |
| Earlier violent self-harm=TRUE (n=153) | 136 (88.9 %) | 126 (57.3 %) | 41.65 | 1.10E-10 |
| First self-harm=TRUE (n=112) | 49 (43.8 %) | 213 (81.6 %) | 51.94 | 5.70E-13 |
| Other psychosis (n=187) | 87 (46.5 %) | - | - |  |
| Earlier self-harm=TRUE (n=87) | 51 (58.6 %) | 36 (36 %) | 8.681 | 0.0032 |
| Earlier violent self-harm=TRUE (n=42) | 34 (81 %) | 53 (36.6 %) | 24.05 | 9.40E-07 |
| First self-harm=TRUE (n=100) | 36 (36 %) | 51 (58.6 %) | 8.681 | 0.0032 |

Supplementary table 3. Subjects born in 1990 or later.

| Variable (subjects born after 1989-12-31) | SZ | SZA | BD | PD | ONAP | All |
| --- | --- | --- | --- | --- | --- | --- |
| Suicide attempt | 154 (34 %) | 50 (39.7 %) | 100 (50.3 %) | 56 (58.3 %) | 68 (26.1 %) | 428 (37.7 %) |
| Self-harm episode | 91 (20.1 %) | 33 (26.2 %) | 58 (29.1 %) | 38 (39.6 %) | 29 (11.1 %) | 249 (21.9 %) |
| Rep. multiple suicide attempts | 74 (16.3 %) | 29 (23 %) | 64 (32.2 %) | 39 (40.6 %) | 30 (11.5 %) | 236 (20.8 %) |
| Multiple self harm episodes | 37 (8.2 %) | 16 (12.7 %) | 34 (17.1 %) | 21 (21.9 %) | 11 (4.2 %) | 119 (10.5 %) |
| For those who report suicide attempts, number reported | 4 (2) | 3 (8) | 3 (2) | 3 (2) | 3 (1.8) | 3 (3) |
| For those who report suicide attempts, number of episodes | 2 (4) | 2 (4) | 2 (3) | 2.5 (4) | 1 (2) | 2 (3) |
| Violent self-harm episode | 35 (7.7 %) | 11 (8.7 %) | 20 (10.1 %) | 16 (16.7 %) | 12 (4.6 %) | 94 (8.3 %) |
| Self harm-episode given reports suicide attempt | 67 (43.5 %) | 28 (56 %) | 51 (51 %) | 36 (64.3 %) | 24 (35.3 %) | 206 (48.1 %) |
| Reports suicide attempt given self-harm episode | 67 (73.6 %) | 28 (84.8 %) | 51 (87.9 %) | 36 (94.7 %) | 24 (82.8 %) | 206 (82.7 %) |
| Multiple self harm episodes given reports multiple suicide att. | 26 (35.1 %) | 13 (44.8 %) | 26 (40.6 %) | 16 (41 %) | 7 (23.3 %) | 88 (37.3 %) |
| Reports multiple suicide att. given multiple self-harm episodes | 26 (70.3 %) | 13 (81.2 %) | 26 (76.5 %) | 16 (76.2 %) | 7 (63.6 %) | 88 (73.9 %) |
| 6 | 26 (16.9 %) | 10 (20 %) | 19 (19 %) | 15 (26.8 %) | 9 (13.2 %) | 79 (18.5 %) |
| Reports suicide attempt given violent self-harm episode | 26 (74.3 %) | 10 (90.9 %) | 19 (95 %) | 15 (93.8 %) | 9 (75 %) | 79 (84 %) |
| Suicidal thoughts past 12 mo | 198 (43.7 %) | 70 (55.6 %) | 119 (59.8 %) | 81 (84.4 %) | 129 (49.4 %) | 597 (52.6 %) |
| Suicidal thoughts ever | 328 (72.4 %) | 106 (84.1 %) | 168 (84.4 %) | 95 (99 %) | 196 (75.1 %) | 893 (78.7 %) |
|  |  |  |  |  |  |  |
| Age | 25 (4) | 25 (4) | 24 (4) | 22 (5) | 23 (4) | 24 (4) |
| No.hospitalizations | 4 (6) | 4 (6) | 3 (5.5) | 4 (7.2) | 2 (2) | 3 (5) |
| Female | 167 (36.9 %) | 77 (61.1 %) | 129 (64.8 %) | 61 (63.5 %) | 108 (41.4 %) | 542 (47.8 %) |
| Working or studying | 82 (18.1 %) | 37 (29.4 %) | 75 (37.7 %) | 28 (29.2 %) | 95 (36.4 %) | 317 (27.9 %) |
| Low education level | 251 (55.4 %) | 53 (42.1 %) | 80 (40.2 %) | 49 (51 %) | 110 (42.1 %) | 543 (47.8 %) |
| Living unsupervised | 284 (62.7 %) | 92 (73 %) | 157 (78.9 %) | 71 (74 %) | 216 (82.8 %) | 820 (72.2 %) |
| Reports bullying | 264 (58.3 %) | 86 (68.3 %) | 131 (65.8 %) | 69 (71.9 %) | 160 (61.3 %) | 710 (62.6 %) |
| Reports physical abuse | 112 (24.7 %) | 32 (25.4 %) | 64 (32.2 %) | 39 (40.6 %) | 63 (24.1 %) | 310 (27.3 %) |
| Reports sexual abuse | 50 (11 %) | 23 (18.3 %) | 41 (20.6 %) | 31 (32.3 %) | 32 (12.3 %) | 177 (15.6 %) |
|  |  |  |  |  |  |  |
|  |  |  |  |  |  |  |

*Supplementary table 4. Pairwise associations for episodes of self-harm*

| Self-harm episodes | Violent self-harm | | | | Followed by another self-harm episode | | | |
| --- | --- | --- | --- | --- | --- | --- | --- | --- |
|  | Factor present | Factor absent | χ^2^ | p-value | Factor present | Factor absent | χ^2^ | p-value |
| All (n=3365) | 659 (19.6 %) | - | - | - | 2000 (59.4 %) | - | - | - |
| Schizophrenia (n=1331) | 299 (22.5 %) | 360 (17.7 %) | 11.3 | <0.0001 | 709 (53.3 %) | 1291 (63.5 %) | 34.31 | <0.0001 |
| Schizoaffective disorder (n=406) | 62 (15.3 %) | 597 (20.2 %) | 5.147 | 0.023 | 216 (53.2 %) | 1784 (60.3 %) | 7.15 | 0.0075 |
| Bipolar disorder (n=1068) | 123 (11.5 %) | 536 (23.3 %) | 63.9 | <0.0001 | 726 (68 %) | 1274 (55.5 %) | 46.83 | <0.0001 |
| Psychotic depression (n=373) | 125 (33.5 %) | 534 (17.8 %) | 50.68 | <0.0001 | 262 (70.2 %) | 1738 (58.1 %) | 19.82 | <0.0001 |
| Other psychosis (n=187) | 50 (26.7 %) | 609 (19.2 %) | 5.963 | 0.015 | 87 (46.5 %) | 1913 (60.2 %) | 13.13 | 0.00029 |
| Earlier self-harm (n=1999) | 458 (22.9 %) | 201 (14.7 %) | 34.1 | <0.0001 | 1486 (74.3 %) | 514 (37.6 %) | 452 | <0.0001 |
| Earlier violent self-harm (n=696) | 377 (54.2 %) | 282 (10.6 %) | 663.6 | <0.0001 | 574 (82.5 %) | 1426 (53.4 %) | 191.9 | <0.0001 |
| First self-harm episode (n=1366) | 201 (14.7 %) | 458 (22.9 %) | 34.1 | <0.0001 | 514 (37.6 %) | 1486 (74.3 %) | 452 | <0.0001 |

Supplementary table 5. Outliers in number of multiple self-harm episodes

| Variable | N, pct / median, IQR |
| --- | --- |
| Diagnosis  SZ  SZA  BD  PD  ONAP | 18 (36.7 %)  7 (14.3 %)  17 (34.7 %)  5 (10.2 %)  1 (2.0 %) |
| Suicide attempt | 46 (93.9 %) |
| Self-harm episode | 49 (100 %) |
| Rep. multiple suicide attempts | 43 (87.8 %) |
| Multiple self harm episodes | 49 (100 %) |
| For those who report suicide attempts, number reported | 5.5 (17) |
| For those who report suicide attempts, number of episodes | 15 (9.5) |
| Ratio of self-harm episodes to reported suicide attempts | 2.2 (5.2) |
| Violent self-harm episode | 26 (53.1 %) |
| Self harm-episode given reports suicide attempt | 46 (100 %) |
| Reports suicide attempt given self-harm episode | 46 (93.9 %) |
| Multiple self harm episodes given reports multiple suicide att. | 43 (100 %) |
| Reports multiple suicide att. given multiple self-harm episodes | 43 (87.8 %) |
| Violent self-harm episode given reports suicide attempt | 25 (54.3 %) |
| Reports suicide attempt given violent self-harm episode | 25 (96.2 %) |
| Suicidal thoughts past 12 mo | 36 (73.5 %) |
| Age | 31 (20) |
| No.hospitalizations | 19 (28) |
| Female | 40 (81.6 %) |
| Working or studying | 6 (12.2 %) |
| Low education level | 24 (49 %) |
| Living unsupervised | 30 (61.2 %) |
| Reports bullying | 37 (75.5 %) |
| Reports physical abuse | 14 (28.6 %) |
| Reports sexual abuse | 19 (38.8 %) |

Supplementary table 6. Cox proportional hazards model of self-harm after discharge from psychiatric hospitalization, main model, excluding outlier individuals in multiple self-harm

| Variable | Hazard ratio | 95% CI | |  | p-value |
| --- | --- | --- | --- | --- | --- |
| Schizoaffective disorder | 1.39 | 1.12 | – | 1.71 | 0.00225 |
| Bipolar disorder | 1.68 | 1.39 | – | 2.02 | 8.68E-08 |
| Psychotic depression | 2.71 | 2.1 | – | 3.5 | 1.80E-14 |
| Other psychosis | 0.958 | 0.719 | – | 1.28 | 0.771 |
| 2 months or less | 1.19 | 0.948 | – | 1.49 | 0.135 |
| 1 month or less | 1.59 | 1.32 | – | 1.92 | 1.14E-06 |
| 1 week or less | 2.18 | 1.8 | – | 2.64 | 1.17E-15 |
| Female sex | 1.57 | 1.35 | – | 1.83 | 3.47E-09 |
| Low education | 1.37 | 1.18 | – | 1.59 | 3.33E-05 |
| Age at discharge | 0.962 | 0.956 | – | 0.968 | 2.20E-34 |
| Sexual abuse | 1.07 | 0.9 | – | 1.28 | 0.431 |

Supplementary table 8. Cox proportional hazards model for hospitalization duration within diagnostic categories

Variable HR 95% CI Low 95% CI High p

Schizophrenia

2 months or less 1.07 0.787 1.44 0.683

1 month or less 1.41 1.11 1.79 0.00551

1 week or less 2.06 1.62 2.62 2.82E-09

Schizoaffective disorder

2 months or less 1.38 0.823 2.32 0.222

1 month or less 1.86 1.19 2.91 0.0063

1 week or less 2.79 1.77 4.41 1.04E-05

Bipolar disorder

2 months or less 1.04 0.637 1.68 0.886

1 month or less 1.45 0.947 2.23 0.0874

1 week or less 1.93 1.25 2.99 0.00315

Psychotic depression

2 months or less 1.23 0.6 2.51 0.575

1 month or less 1.24 0.66 2.33 0.505

1 week or less 2.52 1.36 4.67 0.00327

Other psychosis

2 months or less 0.657 0.247 1.75 0.401

1 month or less 1.21 0.583 2.51 0.61

1 week or less 1.39 0.643 2.99 0.405
